# Ozone Treatment Attenuates Neuroinflammation and Alters miRNA Expression in a Rat Model of Post-Traumatic Epilepsy

**DOI:** 10.1007/s11064-026-04695-w

**Published:** 2026-02-20

**Authors:** Hüseyin Demir, Cumaali Demirtas, Hava Yildirim, Ecem Demir, Sezin Kiroglu Uzun, Kubra Sevgin, Hakan Beyaztaş, Eray Metin Güler, Gulam Hekimoglu, Ender Mehmet Coskunpinar, Nafiye Sanlier, Mehmet Yildirim

**Affiliations:** 1 Department of Neurosurgery, Istanbul Health and Technology University, Istanbul, Turkey; 2 Hamidiye Institute of Health Sciences, University of Health Sciences Turkey, Istanbul, Turkey; 3 Department of Medical Biology, Hamidiye Institute of Health Sciences, University of Health Sciences, Istanbul, Turkey; 4 Department of Radiation Oncology, University of Health Sciences Cam and Sakura City Hospital, Istanbul, Turkey; 5 Department of Physiology, Hamidiye Faculty of Medicine, University of Health Sciences, Istanbul, Turkey; 6 Department of Histology and Embryology, Hamidiye International Faculty of Medicine, University of Health Sciences, Istanbul, Turkey; 7 Department of Medical Biochemistry, Hamidiye Faculty of Medicine, University of Health Sciences, Istanbul, Turkey; 8 Department of Medical Biochemistry, Haydarpaşa Numune Health Application and Research Center, University of Health Sciences, Istanbul, Turkey; 9 Department of Medical Biology, Hamidiye Faculty of Medicine, University of Health Sciences, Istanbul, Turkey; 10 Department of Neurosurgery, University of Health Sciences Turkey Istanbul Training and Research Hospital, Istanbul, Turkey

**Keywords:** Ozone, Post-traumatic epilepsy, Dentate gyrus, Hippocampus, miRNA

## Abstract

The aim of this study was to investigate the effects of intraperitoneal ozone therapy in a post-traumatic epilepsy (PTE) model. An in vivo PTE model was established in male Sprague–Dawley rats, which were randomised to control (n = 8), PTE (n = 10), and PTE + Ozone (n = 10) groups. 0.7 mg/kg ozone was administered intraperitoneally for 3 consecutive days. Seizure activity was video recorded for 120 min and evaluated for latency, frequency, duration, and severity. Behavioral assessments of locomotor activity, anxiety, and spatial memory were conducted using open field, elevated plus, and radial arm maze tests on days 4–6 after the first ozone application. Blood and brain tissues were collected for biochemical assays (SUR1, TRPM4, IL-1β, IL-6, TNF-α, TAS, TOS, OSI, thiol–disulfide homeostasis), histological analyses (H&E, Cresyl Violet, and 8-OHdG immunostaining), and qRT-PCR of epilepsy-related miRNAs. Significant differences were observed among the groups for all serum and brain biomarkers (p < 0.001). The PTE group showed marked increases in SUR1, TRPM4, IL-1β, IL-6, TNF-α, TOS, OSI, TT, NT, and DIS levels, accompanied by a decrease in TAS. Ozone treatment partially reversed these changes by reducing cytokine and oxidative stress markers, improving thiol–disulfide balance, and restoring TAS levels. Behavioural testing revealed beneficial effects of ozone, including reduced immobility, fewer errors in the radial arm maze, and increased open-arm exploration. Although seizure severity, latency, and duration were not significantly altered, seizure frequency showed a decreasing trend (*p* = 0.067). Immunofluorescence for 8-OHdG revealed increased hippocampal oxidative DNA damage in the PTE group, which was attenuated following ozone treatment. Analysis of miRNA expression revealed downregulation in the PTE group, whereas ozone treatment resulted in overall upregulation. There was no statistically significant difference between miRNA expression results and the PTE + Ozone group (*p* = 0.056–0.076). Ozone therapy mitigated oxidative stress and inflammation, improved redox homeostasis, enhanced cognitive and locomotor performance, and reduced hippocampal DNA damage in the PTE model. Furthermore, the observed upregulation of specific miRNAs following ozone treatment highlights a potential molecular mechanism contributing to its neuroprotective effects**.**

**Supplementary Information:**

The online version contains supplementary material available at 10.1007/s11064-026-04695-w.

## Introduction

Traumatic brain injury (TBI) represents a pressing global public health concern. In the USA alone, at least 2.5 million people need medical attention annually due to head trauma [1]. Following the development of trauma, primary brain injury initially occurs in the central nervous system. Within hours or days after the primary injury, secondary brain injury develops. The pathophysiology of this secondary injury involves several mechanisms, including neurotransmitter and free radical release, calcium-dependent cellular injury, gene activation, and mitochondrial malfunction, and inflammation[2]. This secondary injury, which is partially preventable, can adversely affect the patient's prognosis.

Against this background, ozone has attracted attention as a therapeutic agent whose biological effects are mediated through controlled oxidative preconditioning rather than direct antioxidant activity. Medical ozone is an allotrope of oxygen composed of three oxygen atoms, making it one of the most reactive forms of oxygen. It exerts strong oxidizing effects on proteoglycans, lipids, bacteria, viruses, and other microorganisms [3]. Ozone rapidly reacts with blood antioxidants such as uric acid, ascorbic acid, and glutathione, inducing mild oxidative stress and thereby stimulating the cellular antioxidant system [4]. Furthermore, ozone has been reported to activate enzymatic systems that enhance cellular resilience by reducing redox-sensitive transcription factors and proinflammatory cytokine levels [5].

Ozone therapy has been used in medicine for over a century, initially as an antiseptic and later across a range of clinical indications using different administration routes and dosing strategies [6]. In musculoskeletal disorders, ozone reduces disc volume by degrading proteoglycans and attenuating inflammation associated with nerve root compression. Beyond these applications, ozone has gained attention in neurological injury due to its ability to induce oxidative preconditioning and modulate redox-sensitive inflammatory pathways [7]. Experimental studies in neonatal hypoxic–ischemic brain injury models demonstrate that ozone therapy reduces neuronal apoptosis and oxidative damage while enhancing endogenous antioxidant responses [8]. Although derived from hypoxic rather than traumatic paradigms, the shared involvement of oxidative stress and inflammation provides a mechanistic rationale for investigating ozone in traumatic brain injury.

A review of the literature indicates that, despite extensive experimental research on pharmacological and antioxidant interventions in traumatic brain injury, the effects of ozone therapy have not yet been specifically investigated. Therefore, we hypothesized that intraperitoneal ozone administration induces controlled oxidative preconditioning, leading to attenuation of post-traumatic oxidative stress and inflammatory signaling. This adaptive response may indirectly modulate redox- and inflammation-sensitive pathways, including SUR1/TRPM4 channel activation and miRNA expression, thereby limiting secondary neuronal injury and reducing susceptibility to epileptogenesis following TBI.

## Material and Methods

### Experimental Animals

The experimental procedure was approved by Hamidiye Local Ethics Committee for Animal Experiments of the University of Health Sciences (Approval No: 25–11). Twenty-eight male Sprague–Dawley rats, 250 ± 20 g, were used in the experiments conducted within the scope of the project. The animals were housed under controlled environmental conditions (21 ± 2 °C temperature, 50–60% humidity) with a maintained 12-h light/dark cycle. The rats were housed in groups of four in a cage and were feed with standard pellet and ad libitum* water*, ensuring adequate access to food and water.

### Administration of Drugs and Chemicals

Pentylenetetrazole (PTZ) was taken from Sigma-Aldrich (St. Louis, MO, USA), and sevoflurane was procured from Piramal Critical Care (USA). PTZ was dissolved in 0.9% NaCl and administered at 2 mL/kg volume. For ozone generation, the Evozone Basic Plus device (Reutlingen, Germany) was used (flow rate: 10 mL/s; concentration range: 0–80 μg/mL). Ozone was collected from the generator into ozone-resistant syringes and immediately administered intraperitoneally to the rats in the respective group at 0.7 mg/kg for three consecutive days [9, 10]. Intraperitoneal administration was selected to ensure reliable systemic delivery of ozone with precise dose control while minimizing procedural stress.

### Experimental Design

The proposed study was based on an experimental PTE model, induced by administering a subconvulsive dose of PTZ following TBI in rats. In the literature on experimental epilepsy models, there exists a variety of PTE models, which differ according to severity and type of trauma induced, the observation period for development of PTE, and the kind of subconvulsive drugs [11]. Some studies were designed to observe the spontaneous development of seizures following trauma, extending over 6 to 12 months [11, 12]. Currently, there is no standardisation of these models. Yet, in a previous study from our laboratory, subconvulsive doses of PTZ were used to standardize the PTE model, and the impact of TBI to the onset of PTE was confirmed. Starting immediately after the induction of the trauma model, ozone was administered intraperitoneally at 0.7 mg/kg dose for three days. The ozone dose was determined based on a previously published study [8]. The third dose of ozone was administered two hours prior to the injection of subconvulsive PTZ.

On the first day, after the rats were anesthetized with 3% sevoflurane, TBI was induced using the weight-drop method. Forty-eight hours later (on day 3), all animals except the control group were subjected to a predefined PTZ escalation protocol consisting of an initial subconvulsive dose of 30 mg/kg, followed by up to two additional doses of 15 mg/kg at 30-min intervals if Racine stage 4–5 seizures were not observed. Seizure activity was recorded for 120 min in total and scored with Racine scale.

**Fig. 1:**
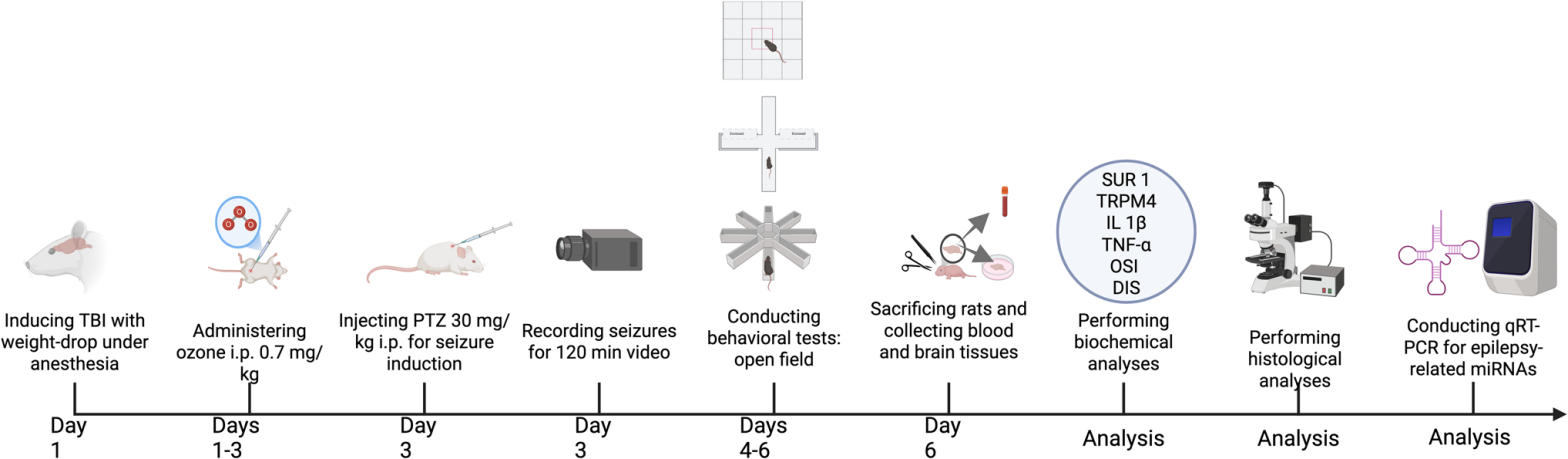
Experimental design of the post-traumatic epilepsy (PTE) model and ozone treatment. TBI was induced in Sprague–Dawley rats using the weight-drop method, followed by intraperitoneal ozone administration (0.7 mg/kg, daily for three days) and PTZ injection (30 mg/kg; additional doses if needed) on Day 3 to establish PTE. Seizure activity was video-recorded for 120 min and scored with Racine scale. Behavioral tests were performed on Days 4–6, and animals were sacrificed for blood and brain collection for biochemical, histological, and molecular analyses. Created in BioRender. (2025) https://BioRender.com/9kkae7p

To assess the effects associated with neuronal damage due to TBI and PTE, as well as the impact of ozone administration, behavioral tests were conducted 24 h after seizure scoring (on day 4). The open field test was executed to assess locomotor condition, and the elevated plus maze was chosen to evaluate anxiety. Radial arm maze test was carried out for spatial memory evaluation over the next three days (days 4, 5, and 6). On the final day of behavioral testing (day 6), the animals were anesthetized with 3% sevoflurane and then sacrificed for the collection of blood and brain tissue samples. Experimental design is depicted visually in Fig. 1**.**

### Experimental Groups

#### Control (n = 8)

The animals in this group had 2 mL/kg of saline intraperitoneally throughout the 3-day study period without undergoing the trauma procedure. Following the behavioral tests, blood and brain tissue samples were collected from the control group.

#### PTE (n = 10)

Beginning immediately after the induction of the trauma model, the animals received 2 mL/kg of physiological saline intraperitoneally for 3 consecutive days. Forty-eight hours after traumatic brain injury, seizures were scored following PTZ at doses of 30 + 15 + 15 mg/kg (2 mL/kg, i.p.). After completion of behavioral tests, blood and brain tissue samples were collected from the PTE group.

#### PTE + Ozone (n = 10)

Starting immediately after the induction of the trauma model, the animals were administered 0.7 mg/kg ozone intraperitoneally for three consecutive days. Forty-eight hours after traumatic brain injury, seizures were scored following PTZ at doses of 30 + 15 + 15 mg/kg (2 mL/kg, i.p.). Following the behavioral tests, blood and brain tissue samples were collected from the PTE + ozone group. The experimental groups are presented in Table 1.

**Table 1:** Experimental Groups

Group	Sample Size (n)	TBI model and Treatment	PTZ Administration	Outcome Measures
Control	8	No trauma model applied 2 mL/kg physiological saline (i.p.) daily for 3 days	Not administered	Behavioral tests (Open Field, Elevated Plus Maze, RAM), Blood and brain tissue collection
PTE	10	TBI model applied 2 mL/kg physiological saline (i.p.) daily for 3 days	PTZ administrated 30 + 15 + 15 mg/kg PTZ (2 mL/kg, i.p.) at 48 h post-trauma	Seizure scoring, Behavioral tests, Blood and brain tissue collection
PTE + Ozone	10	TBI model applied 0.7 mg/kg ozone (i.p.) daily for 3 days	PTZ administrated 30 + 15 + 15 mg/kg PTZ (2 mL/kg, i.p.) at 48 h post-trauma	Seizure scoring, Behavioral tests, Blood and brain tissue collection

### Induction of Traumatic Brain Injury Model

Following anesthesia with 3% sevoflurane, a mild traumatic brain injury was induced by modifying the experimental weight-drop model developed by Marmarou et al. [13–15]. The trauma device has a hollow vertical glass tube attached to a stand. After the rats were anesthetized, each animal was positioned in a prone position on Plexiglas covered with aluminum foil. The rats' head were parallel to the ground along the cranial caudal axis, 1 cm below the glass. TBI was induced by dropping a 250 g steel weight from 100 cm through the tube, directly onto midline of rat's skull. The weight did not make direct contact with the scalp but was dropped onto a 1 cm diameter, 0.3 cm thick steel disc that had been placed on the scalp. To prevent secondary impact, the weight was tethered with a string to control rebound after the initial strike [13, 16].

### Physiological Assessments

#### Induction of Post-Traumatic Epilepsy with Pentylenetetrazole

PTZ was administered 48 h after TBI to assess early post-traumatic seizure susceptibility during the acute secondary injury phase rather than chronic post-traumatic epilepsy. PTE was induced by administering subconvulsive doses of PTZ in three separate injections: 30 + 15 + 15 mg/kg (in a volume of 2 mL/kg, i.p.). In the absence of seizure onset of Racine stage 4 or 5 following the first dose of 30 mg/kg, a second dose of 15 mg/kg was administered 30 min later, followed by a third dose of 15 mg/kg 30 min after the second injection [17]. During this phase of the experiment, video recordings of the animals were obtained, and behavioral seizure activity was evaluated and scored according to the Racine scale.

#### Behavioral Seizure Scoring

As part of the PTE (post-traumatic epilepsy) induction protocol, PTZ was administered in divided doses of 30 + 15 + 15 mg/kg, followed by continuous video recording and behavioral seizure scoring for a total duration of 90 min. Seizure scoring was performed with modified version of the Racine scale [18]. The Seizure severity scale is presented in Supplementary Table 1 [17, 19].

Scoring was conducted in real time, and seizure-related variables were subsequently verified through review of the video recordings. The following behavioral seizure parameters were recorded and used in further analyses: seizure latency, seizure frequency, seizure duration, and seizure severity. Seizure severity was determined as the highest observed on the Racine scale. Seizure latency was time elapsed from the initial 30 mg/kg PTZ dose to the onset of the first seizure reaching stage 4 or 5 on the Racine scale, within the first 30 min. If no stage 4 or 5 seizure occurred following the initial dose, two additional doses (15 mg/kg each) were administered, and the time elapsed until the first stage 4 or 5 seizure was included in the latency calculation. Seizure frequency was the total number of stage 4/5 seizures on the Racine scale during the 90-min post-PTZ injection period. Total seizure duration was cumulative duration of stage 4/5 seizures in this same timeframe. In addition, total PTZ administered to each animal was recorded [17].

#### Behavioral Tests

As part of the behavioral assessments, open field test was chosen to evaluate locomotor status, the radial arm maze test to assess spatial memory performance, and elevated plus maze test was employed for determining anxiety [20–22]. Further specifics about implementation of these tests are given in the supplementary material.

### Biochemical Analyses

#### Collection of Tissue and Serum Samples

Brain tissues obtained from male Sprague–Dawley rats were stored at − 80 °C until analyses. Intracardiac blood were collected into gel-based biochemistry tubes and centrifuged at 3000 × g for 10 min using a Beckman Coulter Allegra® X-30 centrifuge (IN, USA). The serum was aliquoted and stored at − 80 °C.

#### Tissue Homogenization and Total Protein Determination

Tissue samples were homogenized at a ratio of 1:9 (w/v) in 0.1 mol/L phosphate-buffered saline (PBS, pH 7.4) using ceramic beads in a homogenizer for 10 min. Then, the samples were centrifuged at 10.000 × g for 10 min at + 4 °C (Beckman Coulter Allegra® X-30, IN, USA). The total protein content in the resulting supernatant was measured using a commercial kit based on the BCA method (ThermoFisher, 23,225) at a wavelength of 562 nm with a spectrophotometer (BioTek, Synergy™ HTX Multi-Mode Reader with Flash). Protein concentrations were evaluated by comparison with a standard curve.

#### Assessment of Thiol–Disulfide Homeostasis

Thiol–disulfide parameters were measured to evaluate systemic oxidative stress. Total thiol (TT, µmol/L) represents the overall thiol pool, including both native thiols and those oxidized to disulfides. Native thiol (NT, µmol/L) reflects the reduced form of thiols, indicating the antioxidant capacity of serum. Dynamic disulfide levels (DIS, µmol/L) were half of the difference between TT and NT values, representing oxidized fraction of thiols. The percentages of NT/TT, DIS/TT, and DIS/NT were also computed to assess the relative balance between reduced and oxidized thiols. These indices provide a comprehensive picture of redox status, with higher disulfide ratios indicating a shift toward oxidative stress and lower ratios suggesting preserved antioxidant defence [23].All measurements were performed in accordance with manufacturers’ instructions using photometric kits. Measurements were conducted photometrically, and analyte concentrations were calculated using standard curves provided in the kits.

While the results for serum samples were expressed in the units specified by the respective kits, tissue sample results were normalized to total protein content.

The following formulas was used to calculate Oxidative Stress Index (OSI) and Disulfide (DIS) levels:

OSI = [TOS (μmol H₂O₂ equivalent/L) × 100] / [TAS (μmol Trolox equivalent/L)].

DIS = (TT (μM)—NT (μM)) / 2.

#### Analysis of Biochemical Parameters

Serum and tissue samples were analyzed to measure a range of parameters. Total Antioxidant Status (TAS), Total Oxidant Status (TOS), Total Thiol (TT), and Native Thiol (NT) were measured using commercially available photometric kits. Sulfonylurea receptor 1 (SUR1), transient receptor potential cation channel subfamily M member 4 (TRPM4), interleukin-1 beta (IL-1β), interleukin-6 (IL-6), and tumor necrosis factor-alpha (TNF-α) levels were measured using rat-specific ELISA kits.

### Histological Assessments

#### Histological Preparation and Staining

Paraffin-embedded coronal brain Sects. (5 µm; Leica RM2235, Leica Biosystems, Germany) were mounted on poly-L-lysine slides and dried at 37 °C. After deparaffinization in xylene and rehydration through graded alcohols, tissues were stained with Hematoxylin–Eosin (H&E) for general morphology and Cresyl Violet (CV) for neuronal integrity. Slides were dehydrated, cleared in xylene, and mounted with DPX medium (Sigma-Aldrich, USA).

#### Histopathological Evaluation

Neurons in the cortex, hippocampus, and dentate gyrus were assessed for degeneration, necrosis, apoptosis, congestion, inflammation, and hemorrhage. Degenerated or apoptotic neurons were counted in defined microscopic fields; congestion, inflammation, and hemorrhage were graded as mild (1), moderate (2), or severe (3).

#### Immunohistochemistry for 8-OHdG

Oxidative DNA damage was detected by immunostaining for 8-hydroxy-2′-deoxyguanosine (8-OHdG). After citrate buffer antigen retrieval and blocking with normal goat serum, sections were incubated overnight with anti-8-OHdG (Santa Cruz, USA), followed by Alexa 488 secondary antibody (Abcam). Hoechst 33,342 was used for nuclear counterstaining. Images were captured with a Zeiss Axio Vert.A1 fluorescence microscope, and positive cells were quantified using ImageJ (Fiji). Five random fields per section were analyzed, and results expressed as mean 8-OHdG–positive cells per section [24]. Details about histopathological evaluation and immunohistochemistry for 8-OHdG are given in Supplementary Material.

### miRNA Analysis

#### miRNA Isolation and cDNA Synthesis

Total miRNA was extracted from hippocampal tissue using the miRNeasy Micro Kit (Qiagen, Germany). RNA purity and concentration were assessed spectrophotometrically at A260/A280 with a DENOVIX DS-11 FX. cDNA was synthesized using the miRCURY LNA RT Kit (Qiagen). All reagents were kept at − 20 °C and RNA samples at − 80 °C until analysis; RNA concentration was standardized to 100 ng/μl.

#### Quantitative Real-Time PCR (qRT-PCR)

qRT-PCR was performed in triplicate on a Roche LightCycler® 480 II using the miRCURY LNA SYBR Green PCR Kit (Qiagen). U6 snRNA served as the internal control. Specific primers targeted rno-miR-23a-3p, rno-miR-34a-5p, rno-miR-132-3p, rno-miR-134-5p, and rno-miR-324-5p. The thermal protocol included denaturation at 95 °C for 2 min followed by 45 cycles (95 °C × 10 s, 56 °C × 1 min). Amplification specificity was confirmed via melting curve analysis. Details about miRNA analysis are given in Supplementary Material.

### Statistical Analysis

Data distribution normality was assessed with Shapiro–Wilk test. Most variables did not have normal distribution, so Kruskal–Wallis test was applied. For pairwise group comparisons, Mann–Whitney U test was used. Data was expressed as mean ± standard error of the mean (SEM). After Bonferroni correction, p < 0.017 (0.05/3 = 0.01666) was accepted as statistical significance.

For miRNA analysis, data obtained were evaluated using the Qiagen GeneGlobe Data Analysis Center https://geneglobe.qiagen.com/us/analyze), an online software platform for miRCURY LNA miRNA PCR panels. The expression levels of each miRNA were evaluated based on their cycle threshold (Ct) values. Results were normalized for each sample with the housekeeping gene U6 snRNA (ΔCt). Fold changes between groups were calculated as ΔΔCt, and the differential expression of miRNA genes was expressed as 2^ − ΔΔCt (fold regulation, FR). P-values were calculated using repeated 2 − ΔΔCt values for every miRNA in control and experimental groups, applying the Student’s t-test. All graphs were generated using GraphPad Prism software (version 8.0.2).

## Results

### Biochemical Test Results

There were statistically significant differences among the control, PTE, and PTE + ozone groups for all serum biomarkers analyzed (overall comparison for each parameter: p < 0.001; Kruskal–Wallis test). Pairwise group comparisons were performed using Bonferroni-corrected Mann–Whitney U post hoc test (p < 0.017). Compared with the control group, the PTE group exhibited marked increases in serum SUR1 levels (0.56 ± 0.14 vs 3.60 ± 0.65 ng/mL; p < 0.001) and TRPM4 levels (0.27 ± 0.07 vs 2.63 ± 0.84 ng/mL; p < 0.001). Pro-inflammatory cytokine levels were also elevated in the PTE group, including IL-1β (113.8 ± 17.8 vs 521.6 ± 108.9 pg/mL; p < 0.001), IL-6 (266.3 ± 29.8 vs 1166.9 ± 185.6 pg/mL; p < 0.001), and TNF-α (53.6 ± 12.7 vs 472.9 ± 115.0 pg/mL; p < 0.001). Oxidative stress markers were increased, with total oxidative status (TOS) rising from 6.7 ± 1.1 to 15.1 ± 2.4 (p < 0.001), oxidative stress index (OSI) increasing from 13.1 ± 1.7 to 99.1 ± 13.0 (p < 0.001), while total antioxidant status (TAS) decreased from 0.52 ± 0.09 to 0.15 ± 0.03 (p < 0.001).

**Fig. 2:**
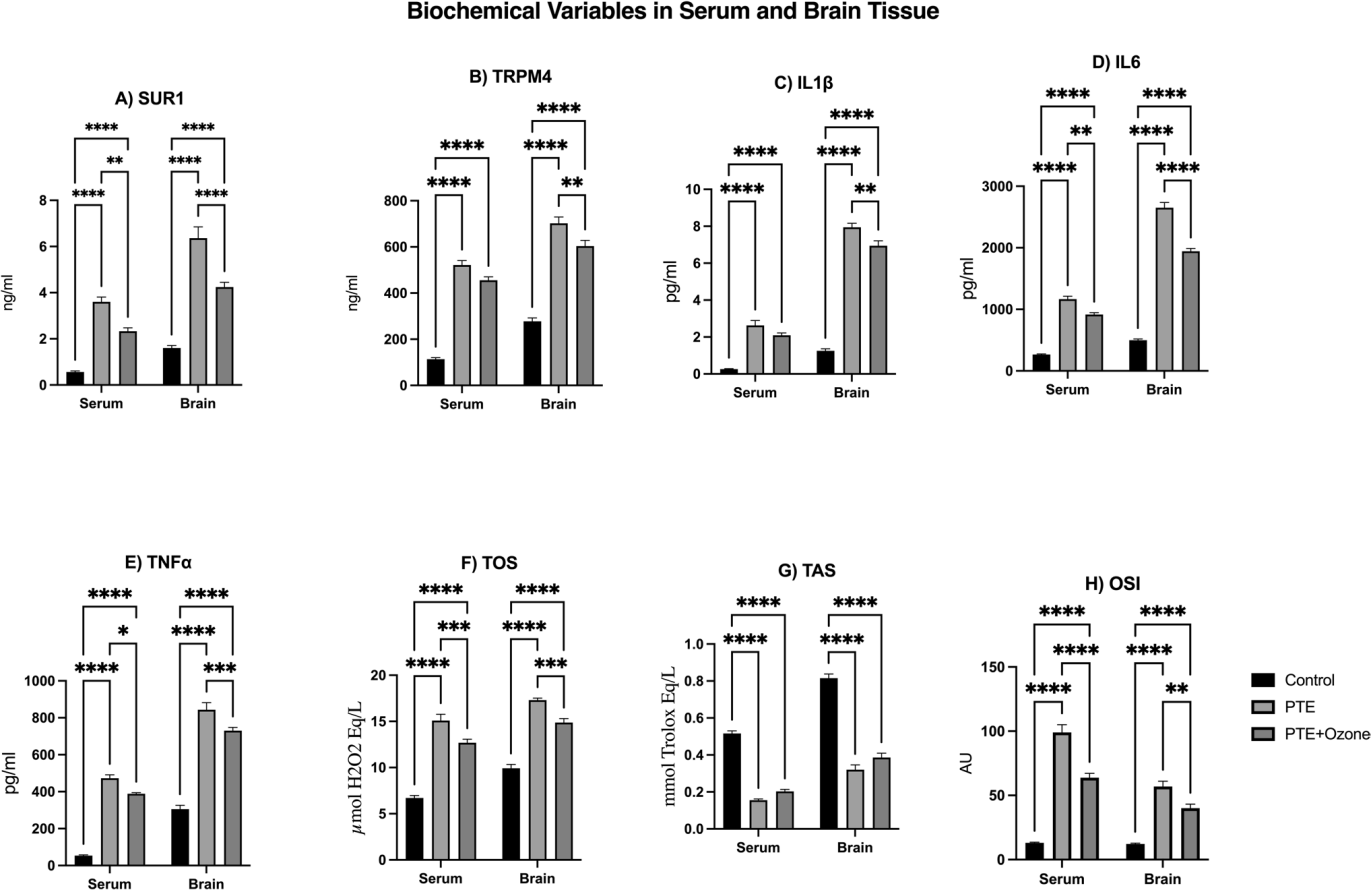
Serum and brain biochemical parameters. Serum and brain concentrations of SUR1 (A), TRPM4 (B), IL-1β (C), IL-6 (D), and TNF-α (E) are given, together with serum oxidative stress markers TOS (F), TAS (G), and OSI (H), presented as mean ± SEM values. SUR1, sulfonylurea receptor 1; TRPM4, transient receptor potential melastatin 4; IL-1β, interleukin-1β; IL-6, interleukin-6; TNF-α, tumor necrosis factor-α; TAS, total antioxidant status; TOS, total oxidant status; OSI, oxidative stress index. Data are given with mean ± SEM. Statistical analyses were performed after the Kruskal-Walli’s analysis of variance, using the Bonferroni-corrected Mann–Whitney U post hoc test for pairwise group comparisons (p < 0.017 (0.05/3 = 0.01666)). *p < 0.017, **p < 0.01, ***p < 0.001, ****p < 0.0001

In the PTE + ozone group, inflammatory and oxidative stress markers were lower than those observed in untreated PTE animals. Serum SUR1 and TRPM4 levels decreased to 2.33 ± 0.45 ng/mL (p < 0.001) and 2.10 ± 0.40 ng/mL (p = 0.151), respectively. Cytokine levels were reduced (IL-1β: 455.5 ± 136.2 pg/mL; p = 0.016, IL-6: 915.8 ± 212.6 pg/mL; p = 0.001, TNF-α: 388.9 ± 143.4 pg/mL; p = 0.001), and oxidative stress markers were partially attenuated (TOS: 12.7 ± 1.8; p = 0.008, OSI: 63.9 ± 10.0; p = 0.001), while TAS increased to 0.20 ± 0.04 (p = 0.002), although values did not fully return to control levels (p < 0.001 for each parameter). Overall, ozone treatment appeared to attenuate both peripheral and central neuroinflammatory processes associated with post-traumatic epilepsy. Quantitative serum and brain biochemical parameters are presented in Fig. 2**.**

### Thiol–Disulfide Homeostasis Results

A significant disruption of thiol–disulfide homeostasis was observed among the control, post-traumatic epilepsy (PTE), and PTE + ozone groups (overall comparison: p < 0.001; Kruskal–Wallis test). Pairwise comparisons were performed Bonferroni corrected Mann–Whitney U post hoc test (p < 0.017). Compared with controls, total thiol (TT) levels were significantly reduced in the PTE group (241.59 ± 7.14 µmol/L vs 424.58 ± 16.10 µmol/L; p < 0.001), as were native thiol (NT) levels (113.15 ± 4.98 µmol/L vs 385.92 ± 8.03 µmol/L; p < 0.001), whereas dynamic disulfide (DIS) concentrations were increased (64.22 ± 3.71 µmol/L vs 21.05 ± 3.99 µmol/L; p < 0.001), indicating enhanced oxidative conversion of thiol groups.

**Fig. 3:**
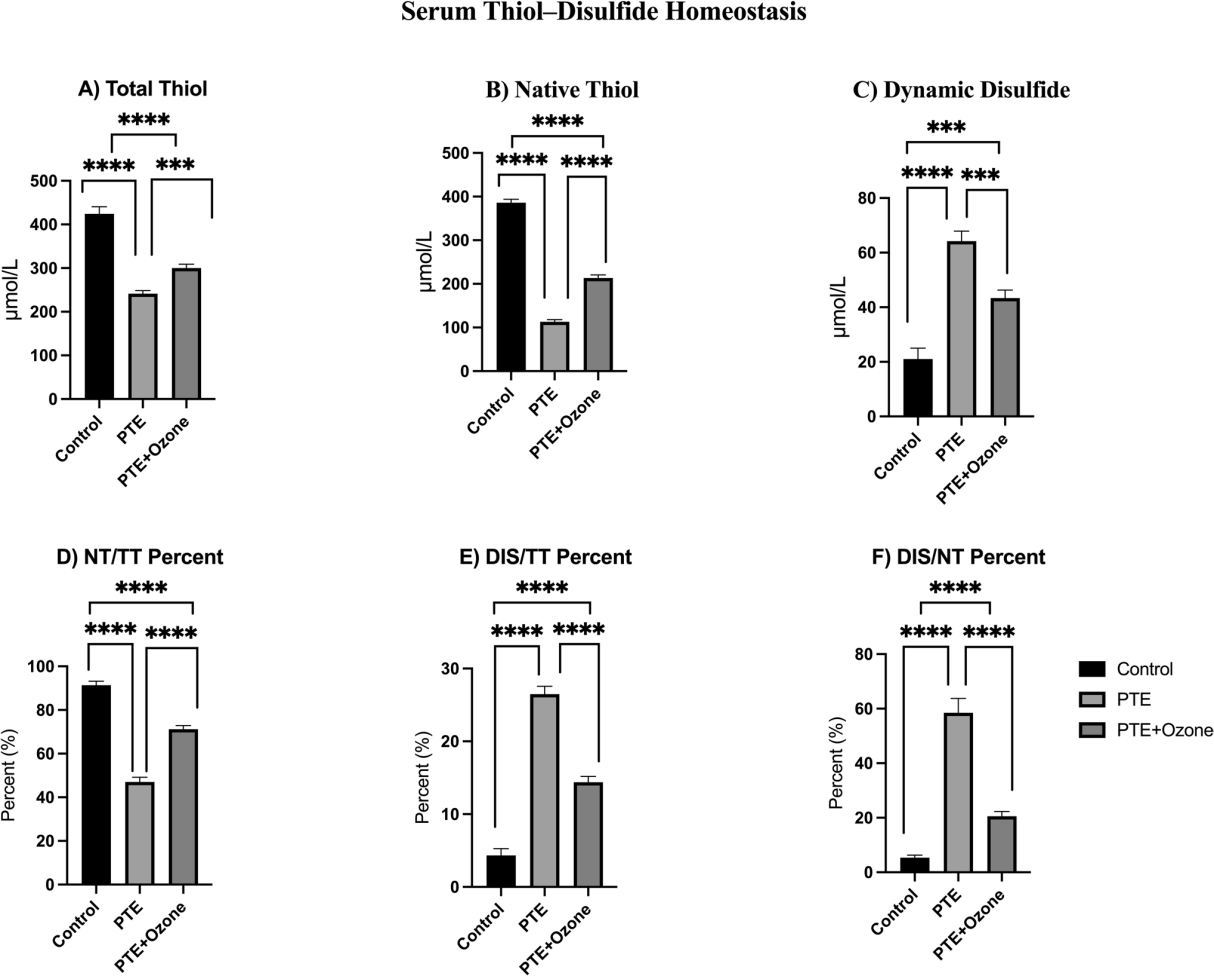
Thiol–Disulfide Homeostasis in Experimental Post-Traumatic Epilepsy (PTE) Model. Comparative analysis of thiol–disulfide parameters among the Control, PTE, and PTE + Ozone groups. (A) Total thiol (TT), (B) native thiol (NT), and (C) dynamic disulfide (DIS) concentrations, as well as (D) NT/TT, (E) DIS/TT, and (F) DIS/NT percentage ratios are shown. The PTE group exhibited significantly reduced total and native thiol levels with concomitant increases in dynamic disulfide and related ratios compared with controls (*p* < 0.05), indicating marked oxidative imbalance. Ozone treatment partially restored thiol concentrations and decreased disulfide levels, suggesting an amelioration of oxidative stress and improved redox homeostasis. Data are given with mean ± SEM. Statistical analyses were performed after the Kruskal-Walli’s analysis of variance, using the Bonferroni-corrected Mann–Whitney U post hoc test for pairwise group comparisons (p < 0.017 (0.05/3 = 0.01666)). *p < 0.017, **p < 0.01, ***p < 0.001, ****p < 0.0001; ns, not significant

Accordingly, the NT/TT ratio decreased (47.03 ± 2.17% vs 91.34 ± 1.86%; p < 0.001), while DIS/TT (26.49 ± 1.09% vs 4.33 ± 0.93%; p < 0.001) and DIS/NT (58.50 ± 5.29% vs 5.34 ± 0.94%; p < 0.001) ratios increased in the PTE group compared with controls. In the PTE + ozone group, TT and NT levels were higher (300.28 ± 8.55 µmol/L; p = 0.001 and 213.60 ± 6.91 µmol/L; p < 0.001, respectively), and DIS levels were lower (43.34 ± 2.96 µmol/L; p = 0.001) than those observed in untreated PTE animals, with corresponding normalization trends in NT/TT (71.23 ± 1.62%; p < 0.001), DIS/TT (14.38 ± 0.81%; p < 0.001), and DIS/NT (20.54 ± 1.73%; p < 0.001) ratios. However, these parameters did not fully return to control values (p < 0.001 for each parameter). These findings demonstrate that ozone administration alleviates the oxidative stress-induced thiol depletion and disulfide accumulation associated with post-traumatic epileptic injury. Quantitative thiol–disulfide homeostasis parameters are presented in Fig. 3**.**

### Physiological Results

#### Behavioral Test Results

**Fig. 4:**
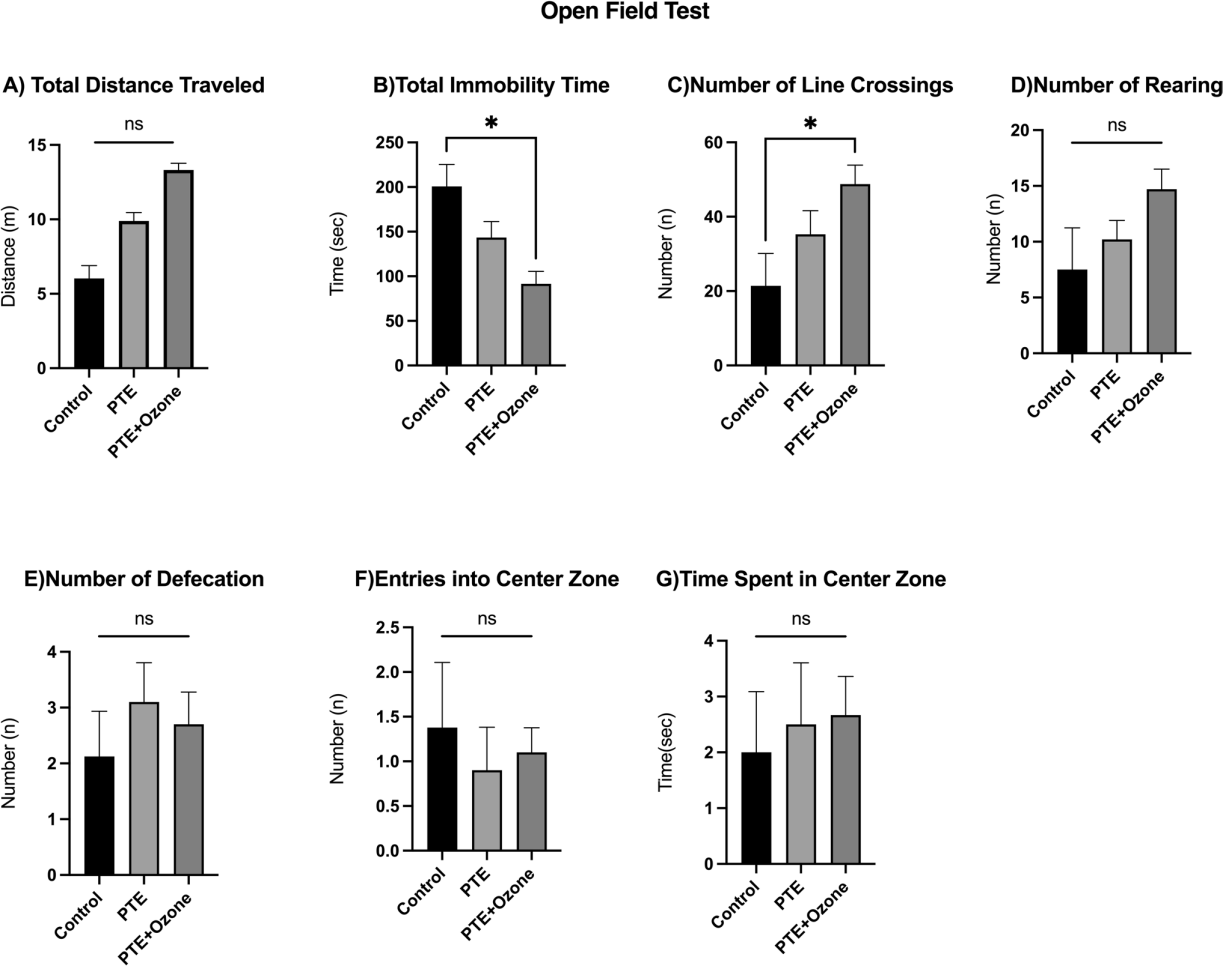
Behavioural parameters in open-field test. The increase in total distance traveled (A) and number of line crossings (C) indicates enhanced locomotor activity. Total immobility time (B) was higher in PTE group compared with control, whereas ozone treatment reduced immobility. A trend toward increased rearing behavior (D) was observed in the PTE and PTE + ozone groups. No significant differences were found in the number of defecations (E), entries into the center zone (F), or time spent in the center zone (G), which are considered indices of anxiety-like behavior. Data are given with mean ± SEM. Statistical analyses were performed after the Kruskal-Walli’s analysis of variance, using the Bonferroni-corrected Mann–Whitney U post hoc test for pairwise group comparisons (p < 0.017 (0.05/3 = 0.01666)). *p < 0.017, **p < 0.01, ***p < 0.001, ****p < 0.0001; ns, not significant

Open-field test outcomes are presented in Fig. 4. There were significant differences among the control, PTE, and PTE + ozone groups for several locomotor parameters (overall comparison: p < 0.05; Kruskal–Wallis test). Pairwise group comparisons were performed using Bonferroni corrected Mann–Whitney U post hoc test (p < 0.017).

Total distance traveled was higher in the PTE group compared with controls (9.89 ± 5.67 vs 6.03 ± 6.90 m; p = 0.09) and was further increased in the PTE + ozone group vs control, but neither increase was statistically significant (13.32 ± 4.57 m; p = 0.02; Fig. 4A). In the PTE group, total immobility time was not statistically significant compared to controls (143.36 ± 57.11 s vs. 200.71 ± 69.32 s; p = 0.09), and ozone therapy was associated with a statistically significant further reduction in immobility time compared to the control group (91.29 ± 44.40 s; p = 0.003; Fig. 4B). Similarly, the number of line crossings was significantly higher in the PTE + ozone group compared with the control group (48.80 ± 16.12 vs 21.38 ± 24.56; p = 0.016; Fig. 4C).

Although no statistically significant differences were detected, a trend toward increased rearing behaviour was observed in both the PTE and PTE + ozone groups compared with controls (Fig. 4D). No significant differences were observed among groups in the number of defecations (Fig. 4E), entries into the center (Fig. 4F), or time spent in the center (Fig. 4G). Overall, these findings suggest that PTE induces motor hyperactivity and increased immobility, whereas ozone treatment partially ameliorates immobility and further enhances locomotor activity without significantly affecting anxiety-related parameters. The increased locomotor activity observed in the open field was interpreted as altered exploratory behavior rather than anxiety or motor impairment, particularly given the early post-traumatic time point and the presence of seizure susceptibility.

**Fig. 5:**
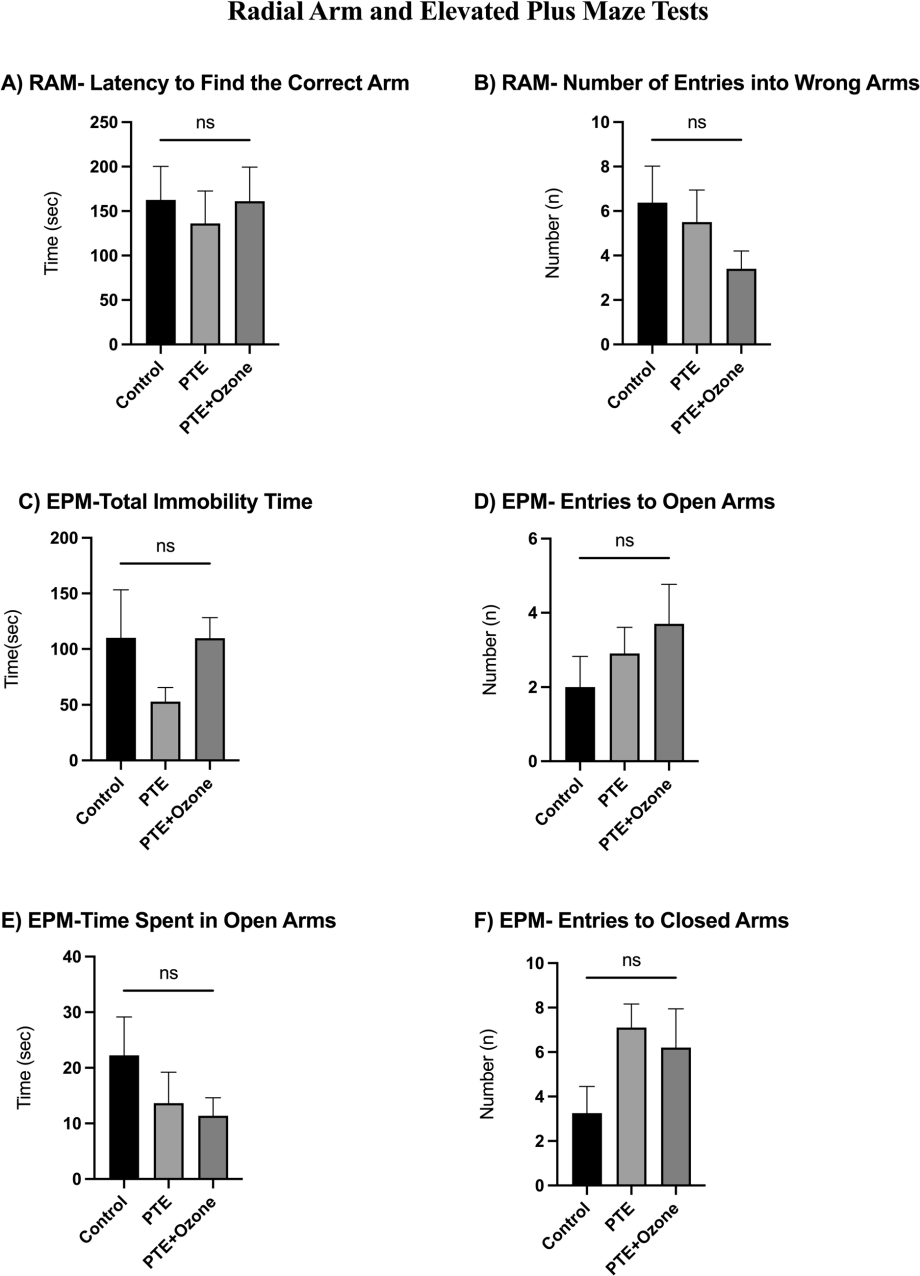
Motor and anxiety-related behavioral parameters. In the radial arm maze test, the latency to locate the correct arm (A) was assessed as a favorable parameter for spatial memory, whereas the number of incorrect arm entries (B) was considered a negative parameter. For elevated plus maze test, total immobility time (C) was evaluated as an indicator of reduced exploratory drive, while the number of open-arm entries (D) and the time spent in open arms (E) were regarded as measures of decreased anxiety-like behavior. The number of closed-arm entries (F) was used for additional parameter of locomotor activity. Data are given with mean ± SEM; Statistical analyses were performed after the Kruskal-Walli’s analysis of variance, using the Bonferroni-corrected Mann–Whitney U post hoc test for pairwise group comparisons (p < 0.017 (0.05/3 = 0.01666)). *p < 0.017, **p < 0.01, ***p < 0.001, ****p < 0.0001; ns, not significant

Behavioral assessments using the radial arm maze (RAM) and elevated plus maze (EPM) did not reveal statistically significant differences among the experimental groups. In the RAM test, latency to find the correct arm and the number of entries into incorrect arms were comparable among the control, post-traumatic epilepsy (PTE), and PTE + ozone groups (p > 0.05 for all comparisons; Fig. 5A–B).

In the EPM test, the number of entries into open arms was similar across groups (control: 2.00 ± 2.33 vs PTE: 2.90 ± 2.23 vs PTE + ozone: 3.70 ± 3.37; p = 0.44; Fig. 5D). Time spent in open arms did not differ significantly (22.26 ± 19.47 vs 13.66 ± 17.57 vs 11.37 ± 10.27 s; p = 0.50; Fig. 5E). Likewise, entries into closed arms were comparable among groups (3.25 ± 3.41 vs 7.10 ± 3.35 vs 6.20 ± 5.51; p = 0.097; Fig. 5F). Overall, no significant group differences were detected in EPM-derived anxiety-related parameters under the tested conditions.

### Findings Related to Seizure

A comparison of seizure parameters between the groups showed no statistically significant differences. Seizure intensity, measured by the Racine score, was similar between PTE group (4.10 ± 0.48) and the PTE + ozone group (3.50 ± 0.76) (*p* = 0.730). First seizure latency tended to be longer in the PTE + ozone group (57.10 ± 10.66 min) compared with the PTE group (43.10 ± 9.91 min; *p* = 0.255). Seizure frequency showed a trend toward reduction following ozone treatment (1.20 ± 0.20 vs. 0.70 ± 0.15; *p* = 0.067). Total seizure duration and total administered PTZ dose was not different between groups (*p* = 0.789 and *p* = 0.411). Comparison of seizure, treatment, and physiological parameters are given in Table 2**.** Body weight on both the first and sixth experimental days was comparable between the groups (*p* > 0.9), indicating that ozone treatment had no effect on overall body weight trajectory. Taken together, these findings suggest that although most seizure parameters remained unchanged, ozone may exert a modest beneficial effect by reducing seizure frequency, warranting further investigation in larger cohorts.

**Table 2:** Comparison of seizure, treatment, and physiological parameters between PTE and PTE + ozone groups

Parameter	PTE (mean ± SEM)	PTE + Ozone (mean ± SEM)	p-value
Seizure Intensity (Racine score)	4.10 ± 0.48	3.50 ± 0.76	0.730
First Seizure Latency (min)	43.10 ± 9.91	57.10 ± 10.66	0.255
Seizure Frequency (n)	1.20 ± 0.20	0.70 ± 0.15	0.067
Total Seizure Duration (sec)	66.50 ± 33.12	45.00 ± 14.85	0.789
Total Administered PTZ Dose (mg/kg)	46.50 ± 4.15	51.00 ± 4.00	0.411
Body Weight – First Day (g)	258.90 ± 13.96	263.70 ± 9.49	1.000
Body Weight – Sixth Day (g)	232.00 ± 11.62	232.30 ± 9.07	0.940

Values are given with mean ± standard error of the mean (SEM). Analysis was performed with Mann–Whitney U test. There was no statistical significance among groups (p > 0.05), although seizure frequency showed a trend toward lower values in the PTE + ozone group (p = 0.067)

### miRNA Results

**Fig. 6:**
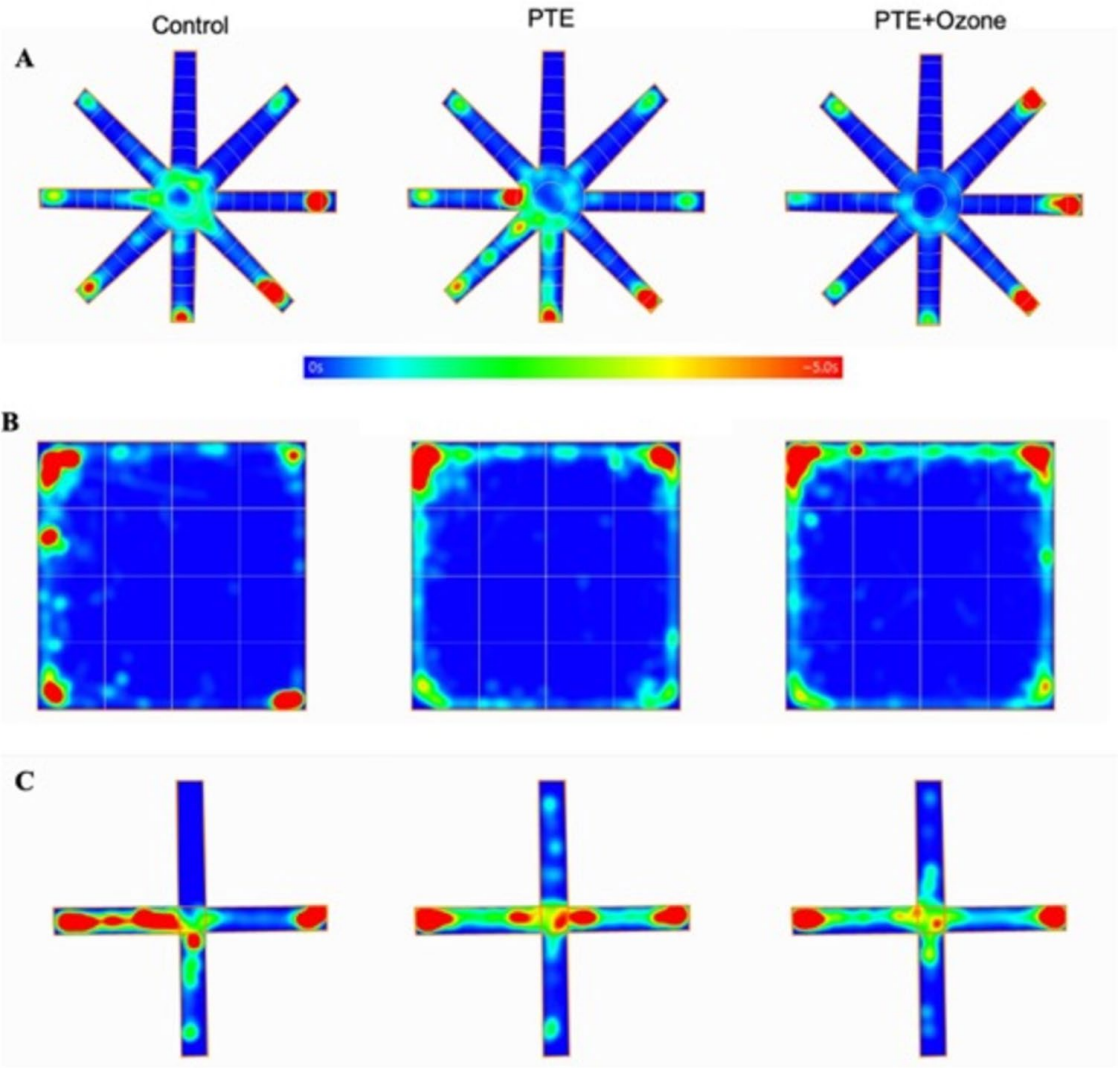
Group average heat maps for treatment. A) Radial arm maze test, B) Open field test, C) Elevated plus maze test. The images are collected from ANYMaze behaviour recording programme. In the diagram, an averaged heat map of the animal’s center point for the group were depicted with the scale given above

**Fig. 7:**
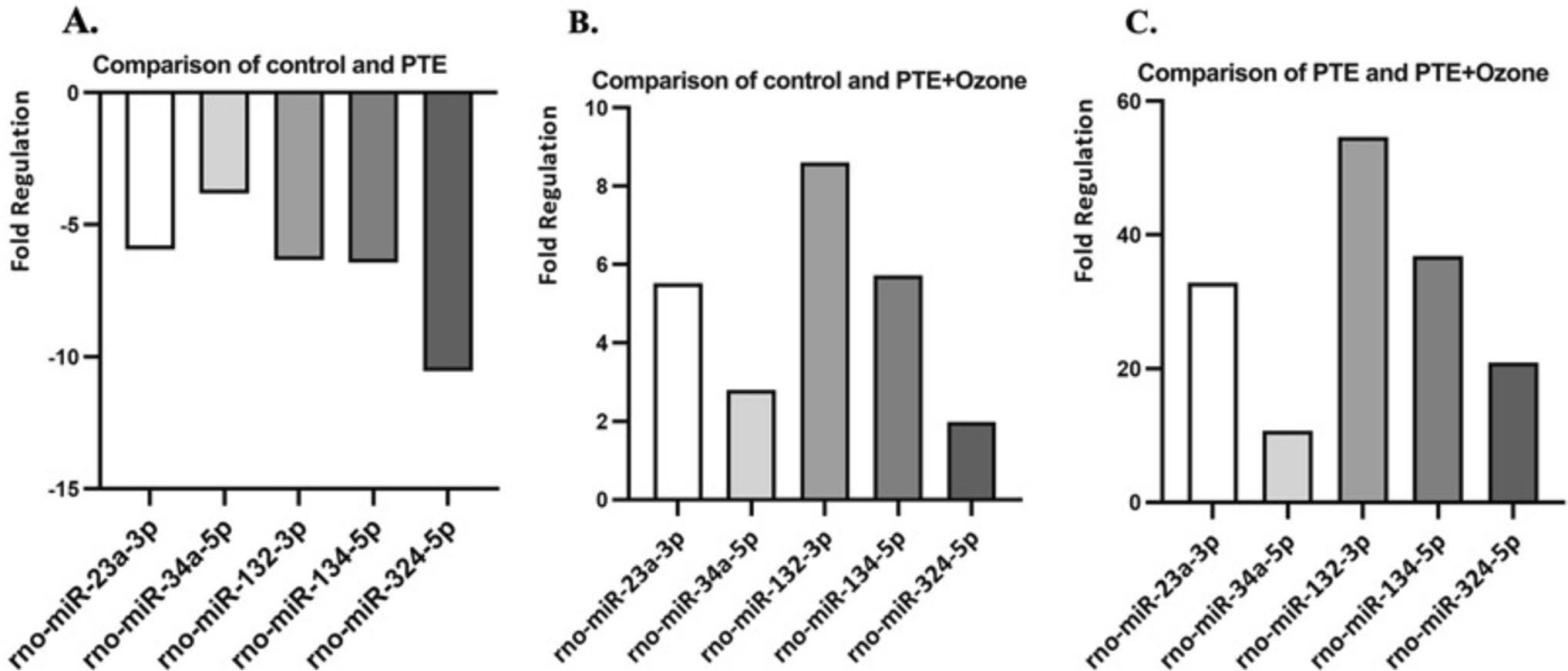
Comparative Fold Regulation of miRNA Expression Between Control, PTE, and PTE + Ozone Groups. A) Comparison between control and PTE groups B) Comparison between control and Ozone treatment group after induction of traumatic brain injury. C) Comparison between PTE and PTE + Ozone groups

Comparative Fold Regulation of miRNA Expression Between control, PTE and PTE + Ozone Groups are given in Fig. 6. When control was compared to PTE group (Figure A), all examined miRNAs (rno-miR-23a-3p, rno-miR-34a-5p, rno-miR-132-3p, rno-miR-134-5p and mmu-miR-324-5p) were downregulated, although these reductions did not reach significance (p > 0.05). Evaluations between control and PTE + Ozone group (Figure B) showed upregulation of all miRNAs, but didn’t reach statistical significance (p > 0.05). Finally, the comparison between the PTE and PTE + Ozone groups (Figure C) demonstrated consistent upregulation of all miRNAs. There was no statistically significant difference between miRNA expression results and the PTE + Ozone group (p = 0.056–0.076). Comparative fold regulation of miRNA are presented in Fig. 7.

PTE induction led to distinct alterations in miRNA expression compared with the control groups, whereas ozone administration in the treatment group partially reversed these changes. This group distribution enabled a direct comparison of baseline, injury, and treatment effects, highlighting the modulatory role of ozone in post-traumatic molecular profiles.

### Histopathology Results

Histopathological scoring demonstrated significant differences between groups across multiple brain regions. In the cerebral cortex, degeneration and necrosis were higher in PTE than control group (p < 0.001). These histological changes significantly decreased with ozone treatment (p < 0.05). While apoptosis showed a near-significant trend (p = 0.051), no significant differences were detected for cortical congestion, inflammation, or hemorrhage (p > 0.05). In the hippocampus, degeneration (p < 0.001), and necrosis (p < 0.001) was increased with PTE which was reversed with ozone treatment.

**Fig. 8:**
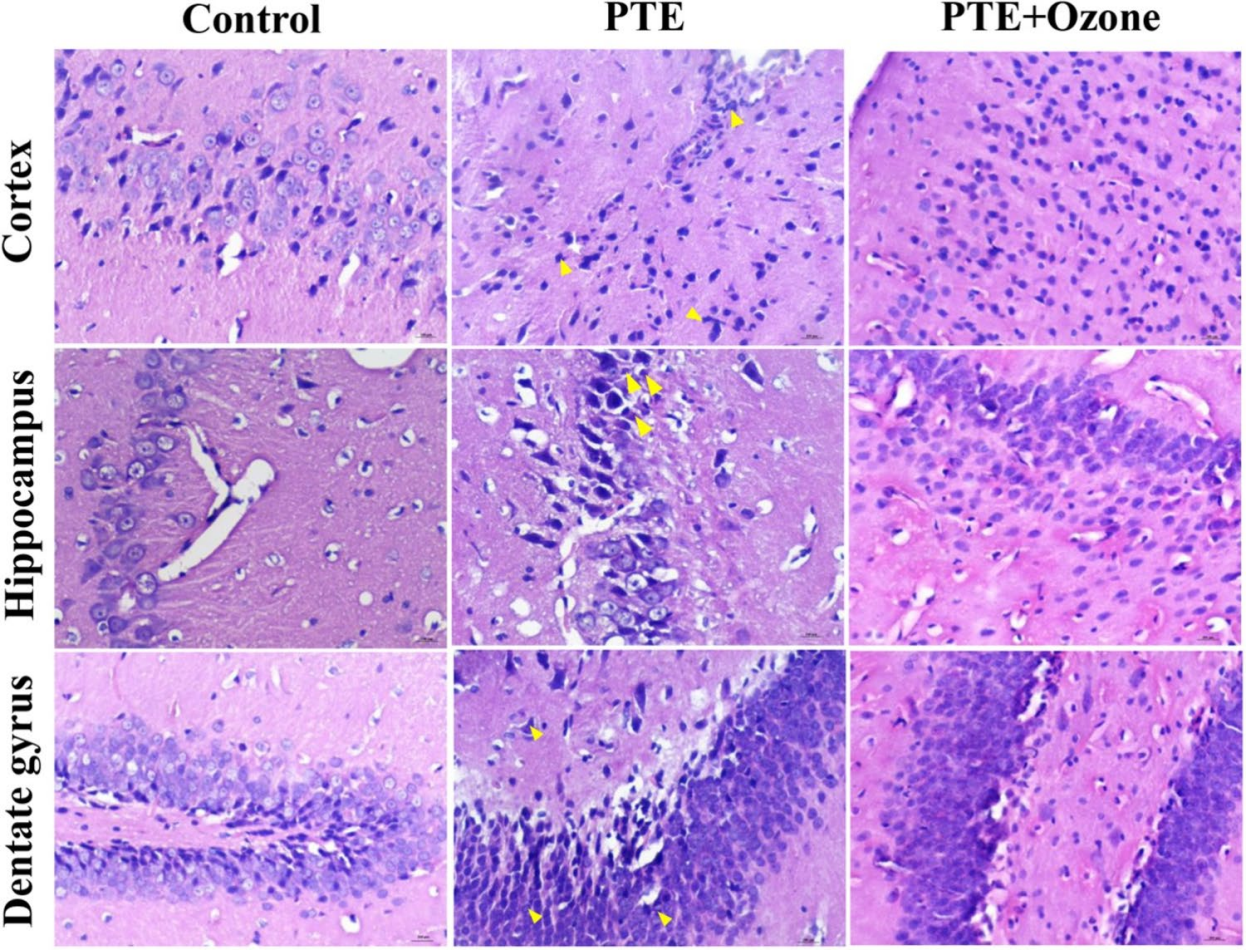
Representative histological images showing apoptosis/single-cell necrosis and neuronal degeneration (yellow arrows) in the superficial cerebral cortex, hippocampus, and dentate gyrus of rats following traumatic brain injury. Left panel: Control rat, showing few or no affected cells throughout the cortex, hippocampus, and dentate gyrus. Middle panel: Traumatic brain injury model rat, with affected neurons displaying degenerative and apoptotic features, including hypereosinophilic cytoplasm, chromatin condensation, and pericellular clearing; classical apoptotic bodies with fragmented nuclei and cytoplasmic blebs were rare. Right panel: Ozone-treated rat, showing a limited number of affected cells throughout the cortex, hippocampus, and dentate gyrus. Staining: H&E; original magnification: 10 ×

Similarly within the dentate gyrus, degeneration was more pronounced in the PTE group (p < 0.001), necrosis showed a borderline increase (p = 0.024), and other parameters—including apoptosis, congestion, inflammation, and hemorrhage—remained comparable between groups (p > 0.05). These changes were mitigated with ozone treatment (p < 0.05). (Table 3**)**. Representative histological images showing apoptosis/single-cell necrosis and neuronal degeneration in the superficial cerebral cortex, hippocampus, and dentate gyrus of rats following traumatic brain injury are given in Fig. 8.

**Table 3:** Histopathological findings in cortex, hippocampus, and dentate gyrus (mean ± SEM)

	**Control**	**PTE**	**PTE + Ozone**
Cortex			
* Deg*	1.00 ± 0.16	*2.03* ± 0.25*	*0.60* ± *0.15†*
* Nec*	0.08 ± 0.06	*0.90* ± 0.15*	*0.13* ± *0.06†*
* Apo*	0.17 ± 0.08	0.33 ± 0.10	0.10 ± 0.06
* Cong*	0.25 ± 0.09	0.67 ± 0.15	0.67 ± 0.09
* Inf*	0.13 ± 0.07	0.30 ± 0.15	0.10 ± 0.06
* Hem*	0.00	0.00	0.00
Hippocampus			
* Deg*	0.63 ± 0.16	*1.57* ± 0.23*	*0.23* ± *0.08†*
* Nec*	0.13 ± 0.07	*0.77* ± 0.15*	*0.10* ± *0.06†*
* Apo*	0.08 ± 0.06	0.07 ± 0.07	0.10 ± 0.06
* Cong*	0.17 ± 0.08	0.63 ± 0.11	0.23 ± 0.08
* Inf*	0.08 ± 0.06	0.20 ± 0.07	0.10 ± 0.06
* Hem*	0.00	0.00	0.00
Dentate Gyrus			
* Deg*	0.63 ± 0.18	*1.60* ± 0.28*	*0.30* ± *0.13†*
* Nec*	0.13 ± 0.09	*0.55* ± *0.17*	*0.10* ± *0.07*
* Apo*	0.19 ± 0.14	0.15 ± 0.08	0.05 ± 0.05
* Cong*	0.06 ± 0.06	*0.80* ± 0.24*	*0.45* ± *0.11*
* Inf*	0.06 ± 0.06	0.10 ± 0.07	0.05 ± 0.05
* Hem*	0.00	0.00	0.00

*Deg* degeneration, *Nec* necrosis, *Apo* apoptosis, *Cong* congestion, *Inf* inflammation, *Hem* hemorrhage. Data are given with mean ± SEM (n = 24–30 for cortex and hippocampus; n = 16–20 for dentate gyrus)

^*^p < 0.017 vs Control; †p < 0.017 vs PTE (Statistical analyses were performed after the Kruskal-Walli’s analysis of variance, using the Bonferroni-corrected Mann–Whitney U post hoc test for pairwise group comparisons (p < 0.017 (0.05/3 = 0.01666))

### Evaluation of 8-OHdG Immunoreactivity

**Fig. 9:**
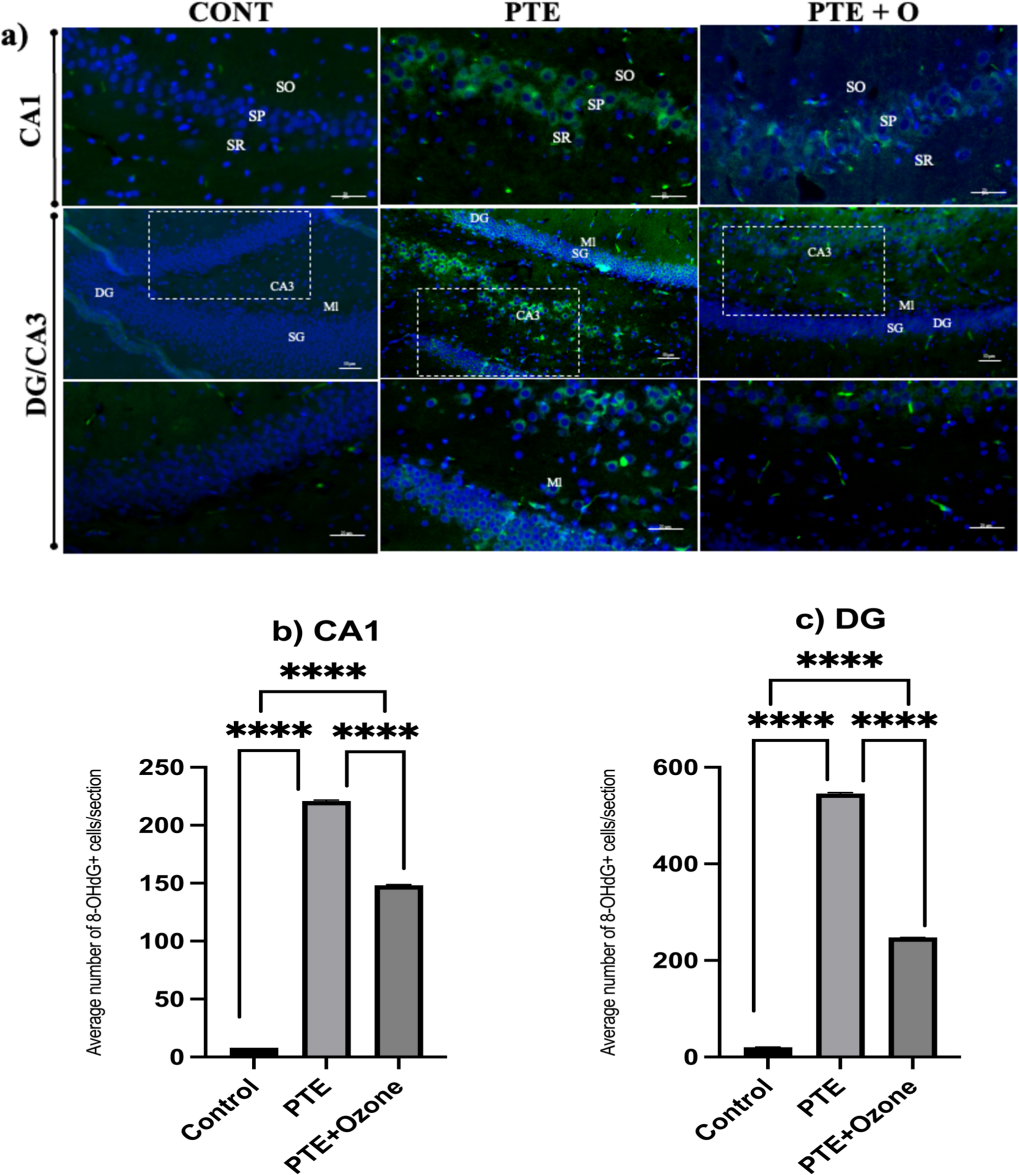
A) Immunofluorescence staining of 8-OHdG in hippocampal regions across the experimental groups. Representative coronal hippocampal sections from control, PTE, and PTE with ozone treatment (PTE + O) groups were stained for 8-hydroxy-2'-deoxyguanosine (8-OHdG; green, Alexa Fluor 488) to assess oxidative DNA damage, and counterstained with Hoechst 33,342 (blue) for nuclear visualization. (A) In the CA1 region, no detectable 8-OHdG immunoreactivity was detected in control group. PTE group displayed strong 8-OHdG staining, particularly in the SP, with additional labelling in the SO and SR. Ozone treatment in the PTE + O group reduced 8-OHdG expression, indicating partial protection against trauma-induced oxidative stress. Magnification: 40 × ; scale bar = 20 μm. In the CA3 and DG regions, no detectable 8-OHdG staining was detected in control group. PTE group had intense 8-OHdG immunoreactivity in the CA3 pyramidal layer and the granule cell layer (SG) of the DG. This staining was markedly reduced in the PTE + O group. Statistical analyses were performed after the Kruskal-Walli’s analysis of variance, using the Bonferroni-corrected Mann–Whitney U post hoc test for pairwise group comparisons (p < 0.017 (0.05/3 = 0.01666)). *p < 0.017, **p < 0.01, ***p < 0.001, ****p < 0.0001; ns, not significant.The lower panel in each group shows a higher-magnification view of the area indicated by the dashed box in the corresponding upper panel of the CA3 region. 8-OHdG: green, Alexa Fluor 488; nuclei: blue, Hoechst 33,342. Upper images: 20 × magnification, scale bar = 50 μm; lower images: 40 × magnification, scale bar = 20 μm. SP: stratum pyramidale SP, SO: stratum oriens, SR: stratum radiatum, Ml: Molecular Layer, DG: Dentate Gyrus, SG: Stratum Granulare, CA1: Cornu Ammonis 1, CA3: Cornu Ammonis 3. Ozone treatment reduced TBI-induced oxidative DNA damage in the CA1 (B) and DG (C) regions, based on quantitative measurement of 8-OHdG staining. Data were given as the mean number of 8-OHdG-positive cells per section

Immunofluorescence analysis of 8-hydroxy-2′-deoxyguanosine (8-OHdG) was performed to evaluate oxidative DNA damage in hippocampal subregions across the control, post-traumatic epilepsy (PTE), and PTE + ozone groups (Fig. 9). In the CA1 region, the number of 8-OHdG–positive cells were low in the control group (8.0 ± 0.0 cells/field), whereas a marked increase was observed in the PTE group (221.0 ± 2.71 cells/field; p < 0.001). Ozone treatment was associated with a reduction in 8-OHdG–positive cells in the CA1 region (148.3 ± 1.49 cells/field) compared with untreated PTE animals (p < 0.001).

Similar patterns were observed in the CA3 region and dentate gyrus (DG). In control animals, 8-OHdG immunoreactivity was minimal (DG/CA3: 20.25 ± 0.71 cells/field). In contrast, the PTE group exhibited significantly increased numbers of 8-OHdG–positive cells in the CA3 pyramidal layer and the granule cell layer of the DG (545.9 ± 5.13 cells/field; p < 0.001). In the PTE + ozone group, oxidative DNA damage was markedly attenuated, with a reduced number of 8-OHdG–positive cells in these regions (247.5 ± 1.35 cells/field) compared with the PTE group (p < 0.001).

Quantitative analysis confirmed increased oxidative DNA damage in the CA1 and DG/CA3 regions of the PTE group relative to controls, with partial attenuation following ozone treatment. Mean ± SD values for 8-OHdG–positive cell counts are presented in Figs. 9B and C. Additional representative images of immunofluorescence staining are provided in Supplementary Figures.

## Discussion

This study investigated the effects of intraperitoneal ozone therapy in an experimental post-traumatic epilepsy (PTE) model. PTE animals demonstrated increased pro-inflammatory cytokines (IL-1β, IL-6, and TNF-α), elevated oxidative stress markers (TOS, OSI), upregulation of ion channel regulators (SUR1, TRPM4), and reduced total antioxidant status (TAS), consistent with a pro-oxidant and pro-inflammatory setting previously implicated in epileptogenesis following TBI [24, 25]. These biochemical alterations are consistent with previous reports implicating neuroinflammation and oxidative stress as central mechanisms in the development of epilepsy following TBI. Ozone administration partially normalized these biochemical alterations, suggesting that its effects may be mediated through restoration redox homeostasis and inflammatory signaling rather than complete normalization of pathological processes [25–27].

Behavioral findings further support this interpretation. TBI-related anxiety-like behavior and spatial memory impairment, commonly reported in experimental and clinical studies [28–30]. Such behavioral changes were partially improved following ozone treatment. However, these behavioral changes occurred in the absence of a statistically significant reduction in seizure severity, latency, or duration, with only a non-significant trend toward reduced seizure frequency. This dissociation indicates that improvement in oxidative and inflammatory parameters alone was insufficient to produce a robust antiepileptic effect, underscoring that ozone should not be considered a stand-alone antiseizure therapy. Instead, the observed behavioral and biochemical effects are more consistent with a supportive or disease-modifying role, potentially enhancing recovery processes associated with secondary brain injury [31–33].

From a translational perspective, intraperitoneal ozone administration represents a controlled experimental approach but does not directly correspond to clinical practice. In humans, ozone autohemotherapy is the most established delivery route, with therapeutic effects attributed to oxidative preconditioning that activates endogenous antioxidant and anti-inflammatory pathways in a dose-dependent manner [34, 35]. In line with this concept, ozone treatment in our model partially mitigated trauma- and PTZ-associated biochemical changes but did not fully restore TAS, particularly in serum, indicating incomplete systemic recovery. Moreover, seizure assessment relied on behavioral scoring without electrophysiological confirmation, limiting conclusions regarding epileptiform activity. These findings highlight the need for cautious extrapolation and emphasize that ozone therapy, if considered clinically, may be most appropriately evaluated as an adjunct to established antiseizure treatments rather than as monotherapy [36, 37].

Histopathological analyses further support a neuroprotective profile of ozone therapy. Ozone-treated animals exhibited fewer degenerating neurons and reduced 8-OHdG immunoreactivity, particularly in hippocampal regions critical for epileptogenesis, suggesting enhanced preservation of neuronal integrity and reduced oxidative DNA damage [38, 39]. In addition to histopathological improvements, we explored whether ozone therapy was associated with alterations in miRNA expression profiles linked to inflammation and neuronal injury.

MiRNAs play a significant role in neuronal apoptosis and the inflammatory response TBI. In a study published in 2020 by Li et al., it was reported that miR-23a-3p has suppressive effects on apoptosis and inflammation [40]. Therefore, mir-23a has been suggested as a therapeutic target that can be used in the treatment of TBI. Although the findings obtained in our study do not express statistical significance, the significant increase in the fold change value of mir-23a-3p because of ozone therapy shows that inflammation will be suppressed, that this gene may be a therapeutic target, and thus supports the findings of Li et al. Inhibition of inflammation is particularly important prognostically [41]. It has also been found that MiR-23a-3p affects the levels of pro-inflammatory cytokines, including IL-1β, IL-10, IL-6, TNF-α, and MCP1, in the TBI mouse model [40]. This result is also consistent with the biochemical findings in our study.

The lack of prognostic biomarkers for PTE is a major obstacle to the development of anti-epileptogenic therapies. In a multicentre study published by Mette Heiskanen in 2025, 23 different expressed miRNAs were reported [42]. Among these genes, rno-miR-132-3p, which is stated to be upregulated in rat plasma on the 2nd day after TBI caused by liquid percussion (FPI), is also a gene included in our study. miR-132-3p is associated with epilepsy-related biological processes such as neuroinflammation, neuronal development, neuroprotection, and neurodegeneration[43]. It plays a significant role in synaptic plasticity, learning and memory, and the maintenance of the blood–brain barrier, all of which are dependent on central nervous system activity. It is not yet clear how neural damage progresses over time and contributes to the development of late-onset seizures. miR-132-3p, which is abundantly expressed in brain cells, is known to be TLR-sensitive and associated with anti-inflammatory signalling[44]. It is known that O3 therapy can protect the brain after acute TBI and facilitate the improvement of neurological behaviours [45, 46]. In this study, we hypothesize that the increase in fold change values of the miR-132-3p gene may be associated with the response to ozone treatment, and that this may occur specifically through the activation of anti-inflammatory signalling pathways by ozone treatment. Although miRNA expression levels did not reach statistical significance, their directional consistency with biochemical and histological findings is noteworthy. Nevertheless, given the absence of prior studies examining ozone-related miRNA modulation in PTE, these findings warrant further investigation in adequately powered studies.

### Limitations

The ozone dose and administration route were based on prior experimental studies; however, no standardized dosing regimen exists for post-traumatic epilepsy, and dose-dependent effects should be interpreted with caution. Because seizure induction relied on a combined traumatic brain injury and subconvulsive pentylenetetrazole paradigm, the independent contributions of trauma and chemoconvulsant exposure cannot be fully separated. In addition, no prospective power calculation was performed due to the lack of pilot effect-size estimates, rendering molecular outcomes—particularly miRNA analyses—exploratory. Finally, behavioral testing was conducted in a fixed sequence, and potential order effects cannot be excluded.

## Conclusion

In this experimental post-traumatic epilepsy model, intraperitoneal ozone administration was associated with partial modulation of oxidative stress–related biochemical parameters and behavioral outcomes. However, seizure frequency was not significantly altered, and electrophysiological confirmation was not performed; therefore, these findings should be considered exploratory. While ozone-induced oxidative preconditioning may influence early post-traumatic epileptogenic processes, further studies incorporating larger cohorts, dose–response analyses, long-term follow-up, and electrophysiological monitoring are required to define its potential role in post-traumatic epilepsy.

## Supplementary Information

Below is the link to the electronic supplementary material.

Supplementary file1 (DOCX 1481 kb)

## Data Availability

No datasets were generated or analysed during the current study.
