# Supplementary material for: Ozone Treatment Attenuates Neuroinflammation and Alters miRNA Expression in a Rat Model of Post-Traumatic Epilepsy
Source: Neurochem Res. 2026 Feb 20;51(2):82. doi: 10.1007/s11064-026-04695-w (PMC12923413; doi:10.1007/s11064-026-04695-w)
Supplement: Supplementary file 1 — Supplementary file1 (DOCX 1481 kb) [file 11064_2026_4695_MOESM1_ESM.docx]

**Supplementary Material**

**Supplementary Table 1.** Seizure severity classification according to the modified Racine scale.

| Stage | Description |
| --- | --- |
| 0 | No response |
| 1 | Ear and facial twitching |
| 2 | Convulsive waves throughout the body |
| 3 | Myoclonic jerks accompanied by rearing |
| 4 | Tonic-clonic seizures with a shift to a lateral posture |
| 5 | Generalized tonic-clonic seizures with loss of postural control |

- 1. **Physiological Assessments**

***Induction of Post-Traumatic Epilepsy with Pentylenetetrazole:*** Forty-eight hours after traumatic brain injury, PTE was induced by administering subconvulsive doses of PTZ in three separate injections: 30 + 15 + 15 mg/kg (in a volume of 2 mL/kg, i.p.). In the absence of seizure onset of Racine stage 4 or 5 following the first dose of 30 mg/kg, a second dose of 15 mg/kg was administered 30 minutes later, followed by a third dose of 15 mg/kg 30 minutes after the second injection[1]. During this phase of the experiment, video recordings of the animals were obtained, and behavioral seizure activity was evaluated and scored according to the Racine scale.

***Behavioral Seizure Scoring:*** As part of the PTE (post-traumatic epilepsy) induction protocol, PTZ was administered in divided doses of 30 + 15 + 15 mg/kg, followed by continuous video recording and behavioral seizure scoring for a total duration of 90 minutes. Seizure scoring was performed with modified version of the Racine scale[2]. The Seizure severity scale is presented in **Supplementary** **Table 1**[1, 3].

Scoring was conducted in real time, and seizure-related variables were subsequently verified through review of the video recordings. The following behavioral seizure parameters were recorded and used in further analyses: seizure latency, seizure frequency, seizure duration, and seizure severity. Seizure severity was determined as the highest observed on the Racine scale. Seizure latency was time elapsed from the initial 30 mg/kg PTZ dose to the onset of the first seizure reaching stage 4 or 5 on the Racine scale, within the first 30 minutes. If no stage 4 or 5 seizure occurred following the initial dose, two additional doses (15 mg/kg each) were administered, and the time elapsed until the first stage 4 or 5 seizure was included in the latency calculation. Seizure frequency was the total number of stage 4/5 seizures on the Racine scale during the 90-minute post-PTZ injection period. Total seizure duration was cumulative duration of stage 4/5 seizures in this same timeframe. In addition, total PTZ administered to each animal was recorded[1].

***Behavioral Tests:*** As part of the behavioral assessments, open field test was chosen to evaluate locomotor status, the radial arm maze test to assess spatial memory performance, and elevated plus maze test was employed for determining anxiety[4-6].

*Open Field Test:*

The open field test apparatus consists of a square arena made of gray Plexiglas material, measuring 100 × 100 cm with a height of 35 cm. This setup is designed to observe the locomotor activity of experimental animals. All tests were conducted between 9:00 AM and 3:00 PM under dim lighting conditions (40 lux). The arena is divided into 16 equal squares, with the central area defined by the four innermost squares. The animals’ behaviors were recorded using a video camera mounted on the ceiling of the apparatus and connected to a computer. Behavioral parameters were recorded and analyzed using an object-tracking software (ANY-maze Video Tracking System, Stoelting Europe, Dublin, Ireland).

Each rat was placed in the open field apparatus starting from a predefined corner, and behavior was recorded for a duration of 5 minutes. The total distance traveled was used as an indicator of locomotor activity. The number of entries into the central area, the time spent in this zone, and the number of defecations were considered indicators of anxiety-related behavior[4-6]. The apparatus was cleaned with 30% alcohol after each trial.

*Radial Arm Maze Test:*

The radial arm maze test apparatus is composed of an eight-arm radial maze and is used to assess spatial learning and memory processes. The experiment consists of three phases: habituation, learning, and testing. In the habituation phase, the rats were placed in the maze for 10 minutes to allow acclimatization. Following this phase, food restriction was implemented. During the learning phase on the following day, food was placed only at the end of one of the eight arms, while the sliding doors of the remaining arms were closed. The rat was placed in the center of the maze, oriented opposite to the baited arm, and allowed to freely enter and feed from the baited arm for 10 minutes.

After this session, food restriction was re-applied, and the test phase was conducted the next day. In this phase, the rat was again placed in the center of the maze, facing away from the previously baited arm, but this time with all arm doors open, allowing free access to all arms. After the rat was placed in the maze, the latency to locate the previously baited arm and the number of incorrect entries into the other arms during this period were recorded.

To eliminate the influence of olfactory cues from previous animals, the entire apparatus was cleaned with 30% ethanol after each session to remove scent traces, defecation, and urination residues. Additionally, researchers conducting the tests took all necessary precautions to minimize potential factors that could influence the animals’ behavior, such as noise or strong odors (e.g., perfume)[4, 5].

All behavioral parameters – excluding the processes of placing and removing the animal from the maze – were recorded and analyzed using object-tracking software (ANY-maze Video Tracking System, Stoelting Europe, Dublin, Ireland).

*Elevated Plus Maze Test:*

The elevated plus maze (EPM) test is a widely used method for assessing anxiety levels in experimental animals. This test is based on the natural tendency of rodents to avoid open and elevated spaces. Anxiety levels are inferred from the amount of time the animal spends in the closed arms of the maze, with increased time in closed arms indicating heightened anxiety.

The EPM apparatus consists of four arms arranged in a plus (+) shape, elevated 60 cm above the ground, and constructed from gray Plexiglas material. Two of the arms are open, while the other two are enclosed. Each arm is 10 cm wide and 50 cm long; the enclosed arms have walls 40 cm in height. The total length of the open arm, including the central intersection area, is 110 cm.

The behavior of the animals placed on the EPM was recorded using an overhead camera connected to object-tracking software (ANY-maze Video Tracking System, Stoelting Europe, Dublin, Ireland). The rats were positioned at the central junction of the maze, facing a predefined open arm. The number of entries into each arm and the time spent in each arm are automatically recorded over a 300-second period using the ANY-maze software.

An increase in closed arm activity (in terms of time and/or entries) was interpreted as an increase in anxiety levels, whereas an increase in open arm activity was interpreted as a decrease in anxiety[4-6]. After each trial, the apparatus was cleaned with 30% ethanol to remove any scent traces or waste from the previous animal.

- 1. **Biochemical Analyses**

***Collection of Tissue and Serum Samples:*** Brain tissues obtained from male Sprague-Dawley rats were stored at −80 °C until analyses. Intracardiac blood were collected into gel-based biochemistry tubes and centrifuged at 3000 × g for 10 minutes using a Beckman Coulter Allegra® X-30 centrifuge (IN, USA). The serum was aliquoted and stored at −80 °C.

***Tissue Homogenization and Total Protein Determination:*** Tissue samples were homogenized at a ratio of 1:9 (w/v) in 0.1 mol/L phosphate-buffered saline (PBS, pH 7.4) using ceramic beads in a homogenizer for 10 minutes. Then, the samples were centrifuged at 10.000 × g for 10 minutes at +4°C (Beckman Coulter Allegra® X-30, IN, USA). The total protein content in the resulting supernatant was measured using a commercial kit based on the BCA method (ThermoFisher, 23225) at a wavelength of 562 nm with a spectrophotometer (BioTek, Synergy™ HTX Multi-Mode Reader with Flash). Protein concentrations were evaluated by comparison with a standard curve.

***Assessment of Thiol–Disulfide Homeostasis:*** Thiol–disulfide parameters were measured to evaluate systemic oxidative stress. Total thiol (TT, µmol/L) represents the overall thiol pool, including both native thiols and those oxidized to disulfides. Native thiol (NT, µmol/L) reflects the reduced form of thiols, indicating the antioxidant capacity of serum. Dynamic disulfide levels (DIS, µmol/L) were half of the difference between TT and NT values, representing oxidized fraction of thiols. The percentages of NT/TT, DIS/TT, and DIS/NT were also computed to assess the relative balance between reduced and oxidized thiols. These indices provide a comprehensive picture of redox status, with higher disulfide ratios indicating a shift toward oxidative stress and lower ratios suggesting preserved antioxidant defence[7].All measurements were performed in accordance with manufacturers’ instructions using photometric kits. Measurements were conducted photometrically, and analyte concentrations were calculated using standard curves provided in the kits.

While the results for serum samples were expressed in the units specified by the respective kits, tissue sample results were normalized to total protein content.

The following formulas was used to calculate Oxidative Stress Index (OSI) and Disulfide (DIS) levels:

OSI = [TOS (μmol H₂O₂ equivalent/L) × 100] / [TAS (μmol Trolox equivalent/L)]

DIS= (TT (μM) - NT (μM) ) / 2

***Analysis of Biochemical Parameters:*** Serum and tissue samples were analyzed to measure a range of parameters. Total Antioxidant Status (TAS), Total Oxidant Status (TOS), Total Thiol (TT), and Native Thiol (NT) were measured using commercially available photometric kits. Sulfonylurea receptor 1 (SUR1), transient receptor potential cation channel subfamily M member 4 (TRPM4), interleukin-1 beta (IL-1β), interleukin-6 (IL-6), and tumor necrosis factor-alpha (TNF-α) levels were measured using rat-specific ELISA kits.

**1.3 Histological Assessments**

***Histological Preparation and Routine Staining Procedures***

Paraffin-embedded coronal brain tissues were sectioned at a thickness of 5 µm using a rotary microtome (Leica RM2235, Leica Biosystems, Germany). The sections were carefully mounted onto poly-L-lysine-coated glass slides and subsequently dried overnight at 37°C to ensure optimal adhesion. Deparaffinization was carried out by immersing the slides in two consecutive changes of xylene for 10 minutes each to completely remove the paraffin. Rehydration was performed through a graded ethanol series, beginning with 100% ethanol (2 changes, 5 minutes each), followed by 96% ethanol (5 minutes) and 70% ethanol (5 minutes), and the slides were finally rinsed in distilled water for 5 minutes to ensure complete hydration. For general histological evaluation, Hematoxylin and Eosin (H&E) staining was applied to visualize the overall tissue architecture and cellular morphology. Additionally, Cresyl Violet (CV) staining, a Nissl stain, was performed to highlight neuronal cell bodies and assess neuronal integrity. Following staining, the slides were dehydrated in a reverse ethanol series (70%, 96%, 100%), cleared in xylene, and coverslipped using a DPX mounting medium (Sigma-Aldrich, USA).

***Histopathological Evaluation***

In all groups, neurons in the cerebral cortex, hippocampus, and dentate gyrus were evaluated using parameters such as degeneration, necrosis, apoptosis, congestion, inflammation, and hemorrhage. Neurons in the cortex and hippocampus that underwent degeneration, necrosis, or apoptosis were counted in three separate fields for each rat. Rats exhibiting congestion, inflammation, or hemorrhage were scored in three separate fields. Scores were given as 1 = mild, 2 = moderate, and 3 = severe. However, neurons in the dentate gyrus that underwent degeneration, necrosis, or apoptosis were counted in two separate fields for each rat. Rats exhibiting congestion, inflammation, or hemorrhage were scored in two separate fields.

***Immunohistochemical Staining for 8-Hydroxy-2'-deoxyguanosine (8-OHdG)***

For the detection of oxidative DNA damage, immunohistochemical staining for 8-hydroxy-2'-deoxyguanosine (8-OHdG) was performed on 5 µm-thick deparaffinized paraffin-embedded coronal brain sections. Antigen retrieval was conducted by incubating the slides in 10 mM citrate buffer (pH 6.0) at 95–98°C for 20 minutes using a water bath. Following antigen retrieval, slides were allowed to cool to room temperature and washed in phosphate-buffered saline (PBS, pH 7.4) for 5 minutes. Endogenous peroxidase activity was quenched by incubating the sections in 3% hydrogen peroxide (H₂O₂) in methanol for 10 minutes. Non-specific binding sites were blocked by incubating the sections with 5% normal goat serum (Vector Laboratories) in PBS containing 0.1% Triton X-100 for 1 hour at room temperature. The sections were then incubated overnight at 4°C with a primary antibody against 8-OHdG (sc-66036, Santa Cruz, Texas, USA). After primary antibody incubation, slides were incubated with a secondary antibody (Alexa 488, ab150113, Cambridge, UK). After washing, the sections were mounted with a mounting medium of PBS- Glycerol (1:1) containing Hoechst 33342 (Thermo Scientific, Massachusetts, USA). Sections that underwent the same procedure without the primary antibody incubation were used as negative controls. All stained sections were examined using a fluorescence microscope (Axio Vert.A1; Carl Zeiss Microscopy GmbH, Jena, Germany) equipped with a digital camera (Axiocam 503 mono; Carl Zeiss Microscopy GmbH, Jena, Germany). Images were acquired using ZEN imaging software (Zeiss) under consistent illumination and exposure settings for all samples.

Fluorescent images were analyzed using ImageJ (Fiji) software (National Institutes of Health, USA) to quantify cells exhibiting 8-hydroxy-2′-deoxyguanosine (8-OHdG) immunoreactivity. Images were separated into individual channels (Hoechst, blue; 8-OHdG, green), converted to 8-bit grayscale, and uniformly contrast-enhanced. A consistent thresholding method was applied to generate binary masks for each channel. Hoechst staining was used to define cell boundaries and ensure that 8-OHdG signals were associated with individual cells. Colocalization of 8-OHdG with Hoechst-labeled cells was determined using the Image Calculator (AND) function, and overlapping particles were quantified. For each tissue section, five randomly selected fields were analyzed, and each field was processed in triplicate to minimize technical variability and ensure reproducibility. Data were expressed as the mean number of 8-OHdG–positive cells per section[8].

- 1. **miRNA Analysis**

***miRNA Isolation and cDNA Synthesis:*** Total miRNA was extracted from hippocampal tissue using the miRNeasy Micro Kit (Qiagen, Germany). RNA purity and concentration were assessed spectrophotometrically at A260/A280 with a DENOVIX DS-11 FX. cDNA was synthesized using the miRCURY LNA RT Kit (Qiagen). All reagents were kept at −20 °C and RNA samples at −80 °C until analysis; RNA concentration was standardized to 100 ng/μl.

***Quantitative Real-Time PCR (qRT-PCR):*** qRT-PCR was performed in triplicate on a Roche LightCycler® 480 II using the miRCURY LNA SYBR Green PCR Kit (Qiagen). U6 snRNA served as the internal control. Specific primers targeted rno-miR-23a-3p, rno-miR-34a-5p, rno-miR-132-3p, rno-miR-134-5p, and rno-miR-324-5p. The thermal protocol included denaturation at 95 °C for 2 min followed by 45 cycles (95 °C × 10 s, 56 °C × 1 min). Amplification specificity was confirmed via melting curve analysis. **Physiological Evaluations**

- 1. **Molecular Analyses (Determination of MicroRNA Levels)**

***Tissue Collection***

Following completion of the behavioral tests conducted in the Department of Physiology on day 6, the animals were anesthetized with 3% sevoflurane and subsequently sacrificed. Cervical dislocation was selected as the method of euthanasia due to its advantages of rapid execution and absence of chemical residues. The brain was carefully removed by dissection, and the cerebral hemispheres were separated at the level of the corpus callosum. The forebrain region of the right hemisphere was placed in Eppendorf tubes and stored at –80 °C until further analysis.

***miRNA Isolation and cDNA Synthesis***

miRNA was isolated from hippocampal tissue using the miRNeasy Micro Kit (Qiagen, Germany; Cat. No. 217084). RNA concentration and purity were determined by spectrophotometric measurements at A260 and A280 wavelengths using a DENOVIX DS-11 FX instrument (USA). For complementary DNA (cDNA) synthesis, the miRCURY LNA RT Kit (Qiagen, Germany; Cat. No. 339340) was employed. Kit reagents were stored at –20 °C until use, while RNA samples were stored at –80 °C. Prior to experimentation, all reagents and RNA samples were thawed on ice. RNA concentrations were standardized to 100 ng/μl.

***Quantitative Real-Time PCR (qRT-PCR)***

Quantitative real-time polymerase chain reaction (qRT-PCR) was performed in triplicate using the Roche LightCycler® 480 II system with the miRCURY LNA SYBR Green PCR Kit (Qiagen, Germany; Cat. No. 339346), following the manufacturer’s protocol. Before initiating qRT-PCR amplification, control PCR assays were conducted to verify the concentration and purity of the cDNA samples.

Comparisons were made between the following groups: control vs. PTE, control vs. ozone, and PTE vs. ozone. U6 small nuclear RNA (U6 snRNA) was used as the housekeeping reference gene. Specific primers were employed for target detection, including rno-miR-23a-3p, rno-miR-34a-5p, rno-miR-132-3p, rno-miR-134-5p, and rno-miR-324-5p. The thermal cycling protocol consisted of an initial denaturation at 95 °C for 2 minutes, followed by 45 cycles of denaturation at 95 °C for 10 seconds and annealing/extension at 56 °C for 1 minute. Amplification specificity was confirmed through melting curve analysis.

1. **RESULTS**

**2.1 Immunofluorescence staining of 8-OHdG Results**


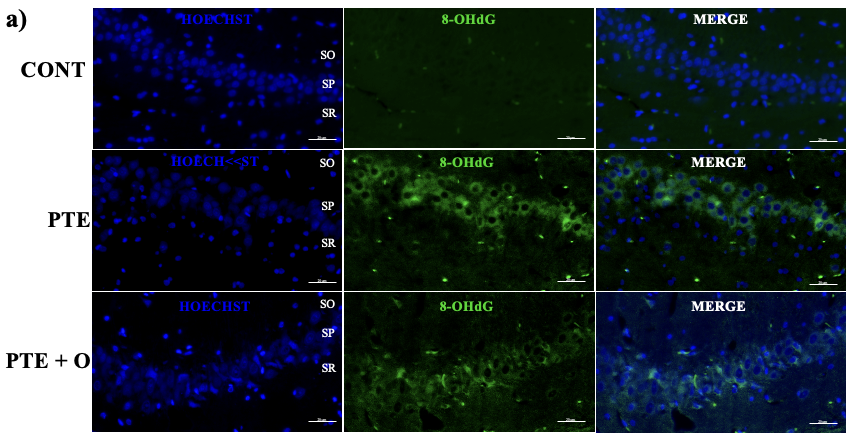


**Supplementary Figure 1**. Immunofluorescence staining of 8-OHdG in the CA1 region of the hippocampus across experimental groups, showing green (8-OHdG) and blue (Hoechst) channels.  CONT: control; PTE: post-traumatic epilepsy; PTE+O: PTE with ozone treatment. SP: stratum pyramidale SP, SO: stratum oriens, SR: stratum radiatum, CA1: Cornu Ammonis 1.


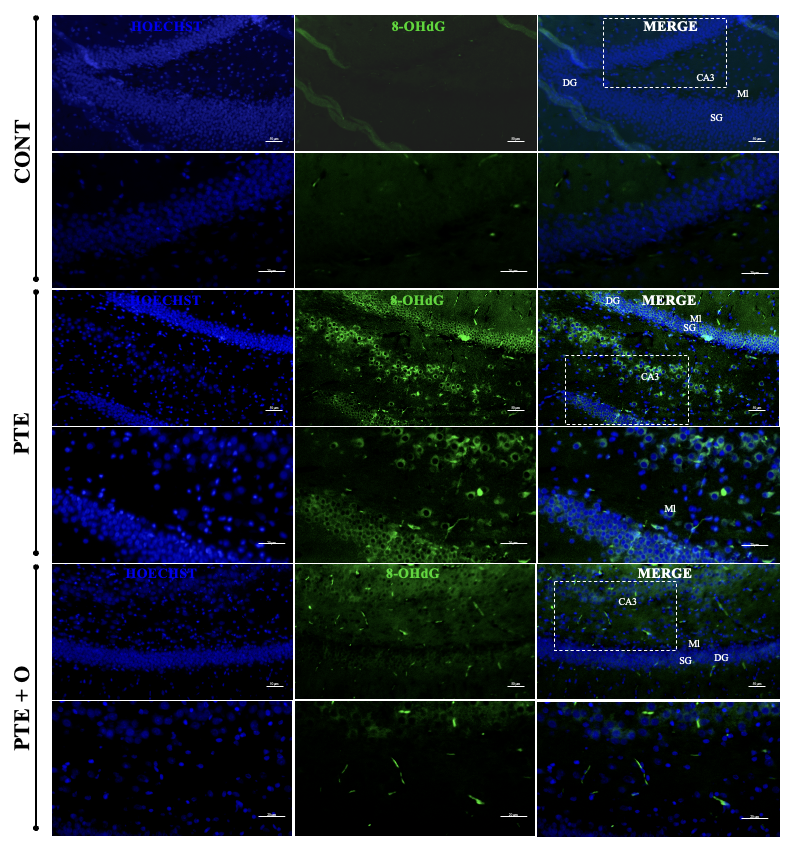


**Supplementary Figure 2.** 8-OHdG Immunofluorescence in the CA3 and DG regions of the hippocampus following traumatic brain injury with green (8-OHdG) and blue (Hoechst) channels.  CONT: control; PTE: post-traumatic epilepsy; PTE+O: PTE with ozone treatment. Ml: Molecular Layer, DG: Dentate Gyrus, SG: Stratum Granulare, CA3: Cornu Ammonis 3.

**References**

1. Efendioglu, M., et al., *Combination Therapy of Gabapentin and N-Acetylcysteine Against Posttraumatic Epilepsy in Rats.* Neurochem Res, 2020. **45**(8): p. 1802-1812.

2. Racine, R.J., *Modification of seizure activity by electrical stimulation: II. Motor seizure.* Electroencephalography and Clinical Neurophysiology, 1972. **32**(3): p. 281-294.

3. Mousavi-Hasanzadeh, M., et al., *The effect of co-administration of pentylenetetrazole with pilocarpine: New modified PTZ models of kindling and seizure.* Pharmacology Biochemistry and Behavior, 2019. **182**: p. 7-11.

4. Aykin, U., et al., *Probiotics Co-administered with LCM Enhance Antiepileptic Efficacy in the Experimental Post-traumatic Epilepsy Model.* Neurochem Res, 2025. **50**(5): p. 290.

5. Demirtas, C., et al., *Effective Protection Against Status Epilepticus Caused by Lithium-Pilocarpine: Combination of Midazolam and Lacosamide.* Brain Behav, 2025. **15**(5): p. e70546.

6. Mazi, A.R., et al., *Extracellular Matrix Alterations Due to Early-Life Adversity: Implications for Auditory Learning in Male Sprague-Dawley Rats.* Mol Neurobiol, 2025. **62**(5): p. 6490-6502.

7. Erel, O. and S. Neselioglu, *A novel and automated assay for thiol/disulphide homeostasis.* Clin Biochem, 2014. **47**(18): p. 326-32.

8. Snyder, B., et al., *Chronic intermittent hypoxia induces oxidative stress and inflammation in brain regions associated with early-stage neurodegeneration.* Physiol Rep, 2017. **5**(9).
